# The affective iconicity of segment and tone in Standard Chinese

**DOI:** 10.3758/s13423-025-02849-5

**Published:** 2026-02-17

**Authors:** Tingting Zheng, Clara C. Levelt, Yiya Chen

**Affiliations:** 1 Leiden University Centre for Linguistics, Leiden University, South Holland, 2311 BE Leiden, The Netherlands; 2 Leiden Institute for Brain and Cognition, Leiden University, South Holland, 2300 RC Leiden, The Netherlands

**Keywords:** Affective iconicity, Segment, Standard Chinese, Tone

## Abstract

While both segmental and suprasegmental aspects of words have been recognised as potential factors influencing their iconic interpretations, how these components collectively drive the associations of form and affective meaning remains elusive. The current study addressed this issue in a lexical tonal language, Standard Chinese, where suprasegmental pitch information distinguishes word meanings. Specifically, we investigated how phonemic information at both the segmental level (i.e., vowels and consonants) and suprasegmental level (i.e., lexical tones) may influence native Standard Chinese listeners’ rating of auditory stimuli’s emotional arousal and valence in two-alternative forced-choice tasks. The results indicated a consistent correlation between tones and the perceived arousal and valence ratings of the tone-carrying nonce words. At the segmental level, consonants were more consistently associated with arousal, while vowels correlated with valence. Furthermore, lexical tones were more influential than segmental phonemes in biasing listeners’ rating of affective meanings. Regarding arousal ratings, nonce words with falling and rising tones tended to be rated with higher arousal than those with high- and low-dipping tones. Additionally, those with an onset /t/ were rated higher in arousal than those with /n/. Regarding valence ratings, nonce words with falling and low-dipping tones were more likely to receive negative ratings than those with high and rising tones. Moreover, stimuli with /u/ were more inclined to be perceived negatively than those with /i/. Though subtle and sporadic, these findings support the universal tendency of affective iconicity across segments and suprasegmental tones.

**Supplementary Information:**

The online version contains supplementary material available at 10.3758/s13423-025-02849-5.

Sound symbolism, the systematic correspondence between sound and meaning in speech, has been proposed as a fundamental property of human languages (Dingemanse et al., 2015; Perniss et al., 2010; Winter et al., 2023), challenging the long-standing assumption that linguistic signs are entirely arbitrary (Hockett, 1960). Contemporary accounts of sound symbolism encompass both *statistical regularities* in sound-meaning associations (e.g., *gl*- in glitter, gleam, glisten) and *iconicity*, which reflects motivated and subjective resemblances between sound and meaning (e.g., high vowel /i/ sounding “smaller”). Statistical regularities are frequency-based while iconicity relies on perceptual analogy (Dingemanse et al., 2015). The present study focuses on iconicity while drawing on insights from statistical regularities where relevant.

Building upon the broader understanding of sound symbolism, previous research has demonstrated robust links between speech sound and meaning (Blasi et al., 2016; Ćwiek et al., 2021; Dingemanse, 2015; Shrum et al., 2012; Svantesson, 2017), including systematic associations between segments and emotional valence (Adelman et al., 2018; Aryani et al., 2018; Auracher et al., 2011; Schmidtke et al., 2014; Whissell, 2003). These affective associations are of particular significance given the central role of emotional communication in human interaction (Darwin, 1872; Davidson et al., 2009) and their relevance for theories of language emergence and evolution (Adelman et al., 2018). Extending this line of inquiry, the present study examines how segments and tones shape affective iconicity in a lexical tone language, providing insights into the perceptual and acoustic mechanisms that underlie affective iconicity (i.e., sound–emotion mappings).

We adopt a dimensional framework that characterises emotions along valence (positive–negative) and arousal (calm–excited) dimensions rather than discrete categories (e.g., joy, fear; Harmon-Jones et al., 2017). This framework has been widely used to describe emotional experiences in studies of iconicity and emotional prosody (e.g., Adelman et al., 2018; Aryani et al., 2018; Bänziger & Scherer, 2005; Laukka et al., 2005).

Distinctive phonetic properties have been shown to help convey emotional arousal and valence. At the segmental level, consonant and vowel phonemes can predict both arousal (German in Aryani et al., 2018; German poetic stanzas in Auracher et al., 2020; Japanese in Kambara & Umemura, 2021) and valence values (German, Polish, Dutch, Spanish, and English in Adelman et al., 2018; Japanese and German in Körner & Rummer, 2023; Dutch and Chinese in Louwerse & Qu, 2017; Chinese and English in Yu et al., 2021). Findings from Spanish, German, English, Chinese, Russian, and Ukrainian seem to point to some cross-linguistic patterns: high front vowels such as /i/ are often linked with positive valence, whereas voiceless plosives /p, t/ and fricatives /s/ tend to occur in words denoting high arousal or negative affect (see also discussion in Calvillo-Torres et al., 2024).

Beyond phonemic segments, prosody has been shown to produce sound-symbolic effects in story reading (e.g., Perlman et al., 2015), demonstrating that suprasegmental cues also contribute to iconicity. Intonational pitch variation (both in terms of average height and contour) has also been consistently associated with emotional arousal and valence (Bänziger & Scherer, 2005; Belyk & Brown, 2014; Kamiloğlu et al., 2020; Laukka et al., 2005; Scherer et al., 2003). Typically, higher global pitch level and wider pitch range are associated with higher arousal and more positive valence (cf. Scherer & Oshinsky, 1977; Stel et al., 2012). Drawing on these studies, Zheng et al. (2024, 2025) examined how lexical tones in Standard Chinese (SC) may contribute to affective iconicity. Their findings indicate that the pitch patterns of lexical tones systematically bias native speakers’ emotional arousal and valence ratings in ways mirroring patterns found in emotional prosody (Zheng et al., 2025). Moreover, by extending phoneme-emotion associations from segmental (Adelman et al., 2018) to also include suprasegmental lexical tone (Zheng et al., 2024), they highlight the importance of considering prosodic cues for insights into language emergence and evolution and for models of affective iconicity.

While affective iconicity has been increasingly documented at both segmental and suprasegmental levels, including lexical tones, the dynamic interactions between these layers of speech remains insufficiently understood. Cross-linguistic findings suggest that segmental and suprasegmental components contribute differently to sound–meaning mappings (Dingemanse et al., 2016; Fort et al., 2015), yet to our knowledge, no well-controlled study has directly compared their effects. We therefore set out to investigate whether, and if so, how consonants, vowels, and lexical tones differ and interact in shaping emotional arousal and valence, as reflected in native speakers’ perceptual judgements.

SC, with its use of suprasegmental pitch variation to distinguish meanings through lexical tones, provides an ideal and necessary testing ground for investigating the interaction of segmental and suprasegmental cues in affective iconicity. SC has four lexical tones, high-level (T1), rising (T2), low-dipping (T3), and falling (T4), which function phonemically like segmental phonemes and are therefore also referred to as tonemes. When produced in isolation, the high-level tone (T1, H) maintains a high pitch level throughout the syllable. The rising tone (T2, R) starts at a mid-pitch level and gradually rises. The low-dipping tone (T3, L) starts at a mid-pitch level, dips, and then often rises again. The falling tone (T4, F) starts at a high pitch level and steeply falls (Zheng et al., 2024).

Based on cross-linguistic findings of phoneme-emotion associations, we selected four widespread segments (/i/, /u/, /n/, and /t/) to examine segmental contributions to affective iconicity. These segments capture key articulatory and acoustic contrasts: front versus back vowels (/i/ vs. /u/) and nasal versus plosive consonants (/n/ vs. /t/). They have also been frequently studied in cross-linguistic iconicity research, providing a solid comparative foundation.

Specifically, the front vowel /i/ has been consistently associated with positive valence across languages including English (Whissell, 2003), German (Adelman et al., 2018; Aryani et al., 2018; Körner & Rummer, 2023), Japanese (Körner & Rummer, 2023), European Portuguese (Garrido & Godinho, 2021), Spanish (Adelman et al., 2018; Calvillo-Torres et al., 2024), and Standard Chinese (Yu et al., 2021). Evidence on arousal is more limited, but /i/ is linked to medium-high arousal in English (Whissell, 2003). In contrast, the back vowel /u/ is linked to negative valence in Japanese and German (Körner & Rummer, 2023), European Portuguese (Garrido & Godinho, 2021), and Standard Chinese (Yu et al., 2021), and limited evidence suggests low arousal associations (Yu et al., 2021). Direct and focused comparisons between /i/ and /u/ remain scarce (Körner & Rummer, 2023).

The nasal consonant /n/ shows variable valence associations across languages: negative in German, Standard Chinese, Russian, and Ukrainian (Adelman et al., 2018; Auracher et al., 2011; Louwerse & Qu, 2017), but positive in Spanish and Dutch (Adelman et al., 2018; Calvillo-Torres et al., 2024; Louwerse & Qu, 2017). This, together with a widespread cross-linguistic bias towards using nasal sounds for body-part terms (e.g., “nose”; Blasi et al., 2016), makes the nasal /n/ a valuable test case for cross-linguistic variation. Finally, the voiceless plosive /t/ is consistently associated with negative valence in German, English, Spanish, and Polish (Adelman et al., 2018; Aryani et al., 2018; Calvillo-Torres et al., 2024) and high arousal (Aryani et al., 2018), providing a clear contrast to /n/.

In SC, there is some evidence on iconicity in segments and tones across various contexts such as onomatopoeia (tones in Thompson, 2018), the perception of size (tones and vowels /i, u, a/ in Chang et al., 2021) and shape (tones and vowels /i, u, a/ in Chang et al., 2021; tones and vowels /i, u/ in Shang & Styles, 2017, 2023), as well as the association with power and gender (tones and frontal vowels in Shih et al., 2019), character traits (tones and consonants /p, t, k/ in Wang, 2021), and preliminary evidence for affective connotations of lexical tones in the general vocabulary (Yao et al., 2013; Yap et al., 2014; Zheng et al., 2024, 2025). These findings suggest the relevance of both phonemic segments (e.g., /i, u, n, t/) and tonemic pitch variations (e.g., T1–T4) in conveying iconic meanings in Chinese.

Limiting our attention to the four chosen phonemes and the four lexical tones in SC enables us to conduct a controlled and systematic investigation of how segmental features and suprasegmental pitch cues interact in affective iconicity, while also situating the findings within a broader cross-linguistic context. We hypothesised that both segmental and suprasegmental components would contribute to affective iconicity in Standard Chinese. Specifically, we expected T4 to be associated with higher arousal and negative valence due to its fast pitch variation and downward contour, while T2 and T1 to be associated with positive valence due to their upward contour and stable high pitch level, and lower arousal due to their less dynamic contours. At the segmental level, we predicted the front vowel /i/ to be associated with positive valence and the back vowel /u/ with negative valence, while the voiceless plosive /t/ was expected to elicit higher arousal than the nasal /n/. Finally, we explored potential interactions between segments and tones on emotional ratings, as well as how vowel identity might modulate perceived arousal, given the limited prior evidence on these combined effects.

## Method

### Sample-size estimation

We conducted a prior sample size estimation using G*Power 3.1 (Faul et al., 2009), setting the odds ratio at 2.5, with an α level of 0.05, a power of 0.80, and assuming no variance explained by other predictors with X parm π set as 0.5. This calculation resulted in a total required sample size of 128 (one-tailed, binomial).

### Participants

A total of 179 native SC-speaking college students participated in the study. All participants had no hearing, visual, speech, or alexithymia disorders and used Standard Chinese for more than 80% of their daily communication. For the final analysis of all the target stimuli, we only included those participants who accurately rated the nontarget validation items (see further details in the Stimuli section). This resulted in a total of 121 participants for the arousal task (aged 18–25 years; 72 women) and 135 participants for the valence task (aged 18–26 years; 84 women).

### Stimuli

#### Auditory stimuli

Two sets of auditory stimuli were used in this study. The first vowel set (V) comprised single vowels /i/ and /u/, each articulated with four SC tones (i.e., /i1/, /i2/, /i3/, /i4/, /u1/, /u2/, /u3/, /u4/). This set investigated the effects of tone, vowel, and their interaction. The second consonant–vowel nonce word set (CVL) combined the consonants /t/ and /n/ with vowels /i/ and /u/ and the coda /l/ to form monosyllabic nonce words (/til/, /nil/, /tul/, /nul/). This set explored the effects of tone, vowel, consonant, and their interactions. An additional (third) set included four disyllabic nonce words with consonant–vowel–consonant–vowel structure (CVCV), which was intended to conceptually replicate a previous study (Zheng et al., 2025) with nonce words so as to better control segmental and tonal compositions and exclude possible effects of lexical meanings. The use of nonce words in iconicity studies (McCormick et al., 2021) and auditory presentation (Cuskley et al., 2017) has often been adopted to bias listeners’ processing towards sound properties rather than semantic meanings, thereby gaining insight into iconicity as a pre-semantic phenomenon (Sučević et al., 2015; Westbury, 2005).

In addition to the target stimuli, we also included two real words *baozao* (/pɑʊ//tsɑʊ/ T4T4, meaning *irritable*) and *xingfen* (/ɕɪŋ//fən/ T1T4, meaning *excited*) as validation items. The arousal and valence ratings for *baozao* have been shown to be consistently negative and high arousing, while those for *xingfen* have been shown to be consistently positive and high arousing (Wang et al., 2008; Xu et al., 2022). Therefore, they were chosen to evaluate participants’ involvement in the online tasks and their deviant inappropriate ratings were used as an objective criterion for participants’ exclusion.

**Fig. 1:**
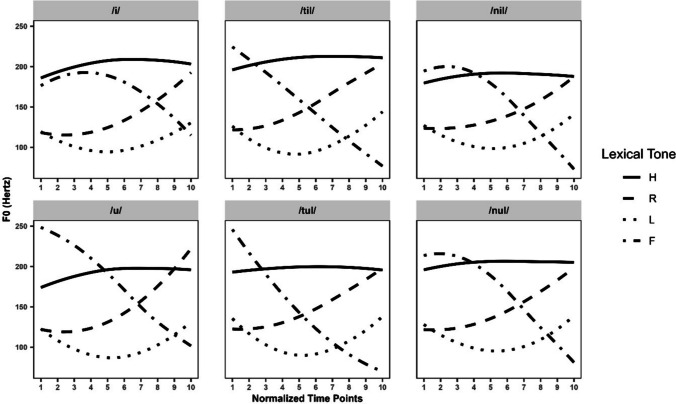
Pitch contours of auditory stimuli

All stimuli were recorded in isolation with a Sennheiser MKH416T microphone (sampling size 44.1 kHz, 16 bit) at Leiden University’s Phonetics Lab by a male native speaker of Standard Chinese. He was born and raised in Beijing and unaware of the study’s purpose. He was asked to produce the stimuli as a statement without any emphasis. The stimuli were recorded three times, with the stimulus list randomised for each recording, and edited in Praat (Boersma & Weenink, 2024). Tokens judged to be most clearly articulated by the first author and another native speaker consultant were selected. Each stimulus was normalised to an average intensity of 70 dB SPL. We segmented the auditory stimuli and set the boundaries of the onset and offset for each syllable. Then, we defined the number of *f0* points (i.e., 10) and *f0* range (i.e., 45–400 Hz) to be extracted from the target rime. The pitch contours of the six tokens (two single vowels and four monosyllabic nonce words) are shown in Fig 1.

#### Visual stimuli

Two pairs of emoji faces representing high vs. low arousal and positive vs. negative valence were selected from the EmojiGrid (Toet & van Erp, 2019). EmojiGrid was developed to facilitate more spontaneous and intuitive communication about affective experiences. Its assessments align well with other self-report tools like SAM and Likert scales and demonstrate the typical V-shaped relationship between arousal and valence.

### Procedure

The experiment was conducted on the Gorilla platform (https://gorilla.sc/). After recruitment, each participant received a personalised link to the experiment, accessible only via a PC or laptop. A headphone test ensured that all participants completed the experiment in a quiet environment using headphones (Milne et al., 2021).

All instructions were given in written Chinese. Participants were first introduced to the target emotional dimensions (arousal and valence) and their corresponding emoji icons through a quiz on assigning emojis to emotional dimensions in specific scenarios. Feedback was provided, and participants could proceed only after correctly completing all assignments to ensure their correct understanding of the emotional dimensions and emojis used in the task.

For both valence and arousal ratings, a two-alternative forced-choice (2AFC) task was employed. In contrast to Likert rating tasks, which are commonly used for familiar or meaningful words (e.g., McLean et al., 2023; Schmidtke & Conrad, 2024; Winter & Perlman, 2021), the 2AFC paradigm is often preferred for unfamiliar or nonce stimuli because it elicits clear binary decisions without overcomplicating the judgment process (e.g., Monaghan et al., 2012).

Participants performed the valence and arousal rating tasks in a counterbalanced order. However, attrition during the task phase resulted in a slight difference in the final distribution of participants across conditions. Specifically, 93 participants started with the valence task, whereas 86 started with the arousal task. Despite this minor imbalance, the counterbalancing procedure was effective in minimising order effects.

Each participant completed a series of five sessions. The sequence included a practice session comprising four trials using unrelated nonce words and single vowels to familiarise participants with the task. Subsequently, participants completed three experimental sessions (i.e., V, C/i/L, C/u/L), each consisting of eight trials. The order of these experimental sessions was counterbalanced across participants using a Latin-square design. The last session, including the CVCV stimuli, followed by the validation items, was presented at the end to minimise potential influences from tone-bearing units and ensure consistent presentation of tones. Notably, the three experimental sessions focused on stimuli with a single tone as the independent variable, whereas the CVCV session involved stimuli with two tones, i.e., tonal sequence patterns (for results, see Zheng et al., 2025). The entire experiment lasted approximately 20 min. Additionally, a mandatory 10-second break was included after each session to minimise potential fatigue. Within each session, stimuli were presented in eight pseudorandomised orders, ensuring alternation between onsets and a minimum distance between identical lexical tones. Each stimulus was played automatically once and could be manually replayed up to five times. Participants indicated their perceived arousal or valence of the auditory stimulus by selecting one of two emojis, which were horizontally presented with their positions counterbalanced across trials.

### Data analysis

Factorial generalised linear mixed models (GLMMs) were employed separately for the single vowel (V) dataset and the nonce word (CVL) dataset to examine the main effects of tone, vowel, and consonant, and their interactions, on arousal and valence choices. These analyses were conducted using the *lme4* (Bates et al., 2014) package in R (R Core Team, 2023). Logistic regression models with a binomial probability distribution and a logit link function were utilised to analyse binary outcomes.

Specifically, four models, i.e., a null model and three alternative models, were fitted for both arousal and valence ratings in the V dataset. These alternatives included fixed effects of (a) tone, (b) vowel, and (c) tone, vowel, and their interaction. In the CVL dataset, eight models were fitted for both arousal and valence ratings, namely a null model and seven alternative models incorporating fixed effects of (a) tone; (b) vowel; (c) tone, vowel, and their interaction; (d) onset consonant; (e) tone, onset consonant, and their interaction; (f) vowel, onset consonant, and their interaction; and (g) tone, vowel, onset consonant, and their interactions. To account for potential participant heterogeneity, we initially included random intercepts and slopes by subject. However, after fitting the models, we encountered convergence issues and overfitting with the random slopes. Therefore, we opted for a simpler model with random intercepts only (by-subject). Overdispersion was assessed using residual ratios, calculated as the sum of squared Pearson residuals divided by the residual degrees of freedom, to evaluate whether the observed variability in the outcomes exceeds what would be expected under the assumed binomial distribution. Main results are reported in the Results section while detailed statistical reports for all fitted models and the corresponding statistical powers are provided in the Supplementary Tables S1.1–4.15. In addition, we assessed the reliability of participant ratings via intraclass correlations, the results of which can be found in Supplementary Table S5.1.

For pairwise multiple comparisons, we utilised the *emmeans* package (Lenth, 2023), applying a Bonferroni correction to control the false positive rate (Type I errors). Post hoc power analyses were performed for each coefficient in all models using the *pwr* package (Champely et al., 2020) to ensure sufficient power to detect the effects of each predictor. These analyses help to support the reliability of our findings by confirming that the sample size was adequate to identify significant effects, thereby reducing the risk of Type II errors.

We used the Odds Ratio (*OR)* as a measurement in our data analysis. Odds represent the ratio of the probability of an event occurring (e.g., high arousal) to the probability of it not occurring (e.g., being rated as low arousal). The *OR* is a statistical measure that compares the odds of an event occurring in one group to the odds of it occurring in another group. For example, an *OR* indicates how much more likely high arousal is to be chosen in one category (e.g., /i/) compared to another category (e.g., /u/).

To visualise the results, mosaic plots were employed to illustrate how the frequency of arousal or valence choices varied with the predictors (Friendly & Meyer, 2015). The tiles were coloured and shaded based on standardised residuals, indicating deviations from expected frequencies under the assumption of independence, where the expected probability is equal for all levels of the categorical variables. Positive residuals denote observed frequencies that are higher than expected, while negative residuals indicate lower-than-expected frequencies. The shading provides information about relative frequencies within the same row. Importantly, standardised residuals in the mosaic plots measure departures from independence but do not directly correspond to the inferential statistics of the fitted models. Instead, they offer additional insight into the strength and direction of the association between factors and emotional dimensions.

## Results

**Fig. 2:**
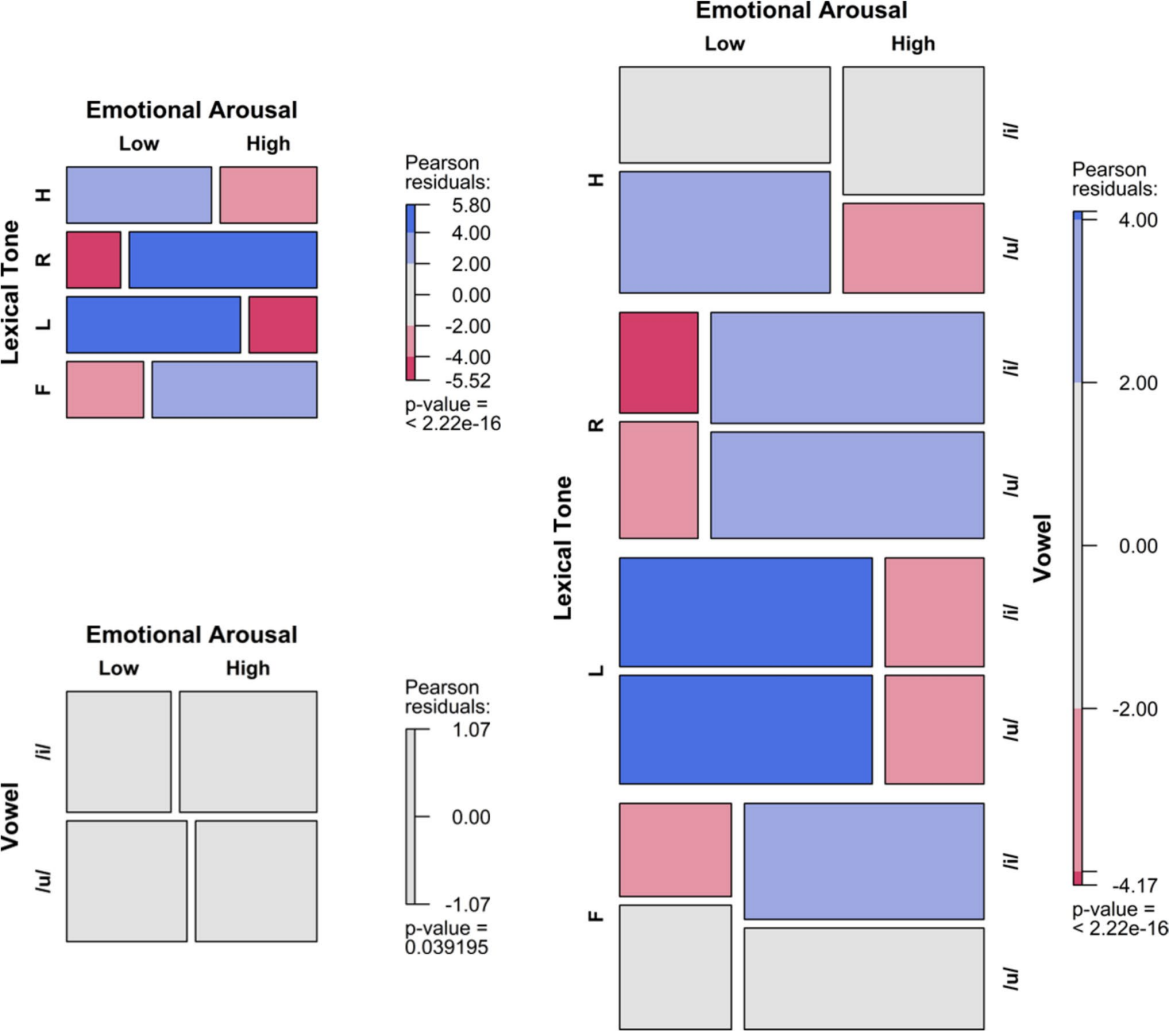
Arousal frequency variations across SC tones, vowels, and their interactions in the V dataset. (Colour figure online)

**Fig. 3:**
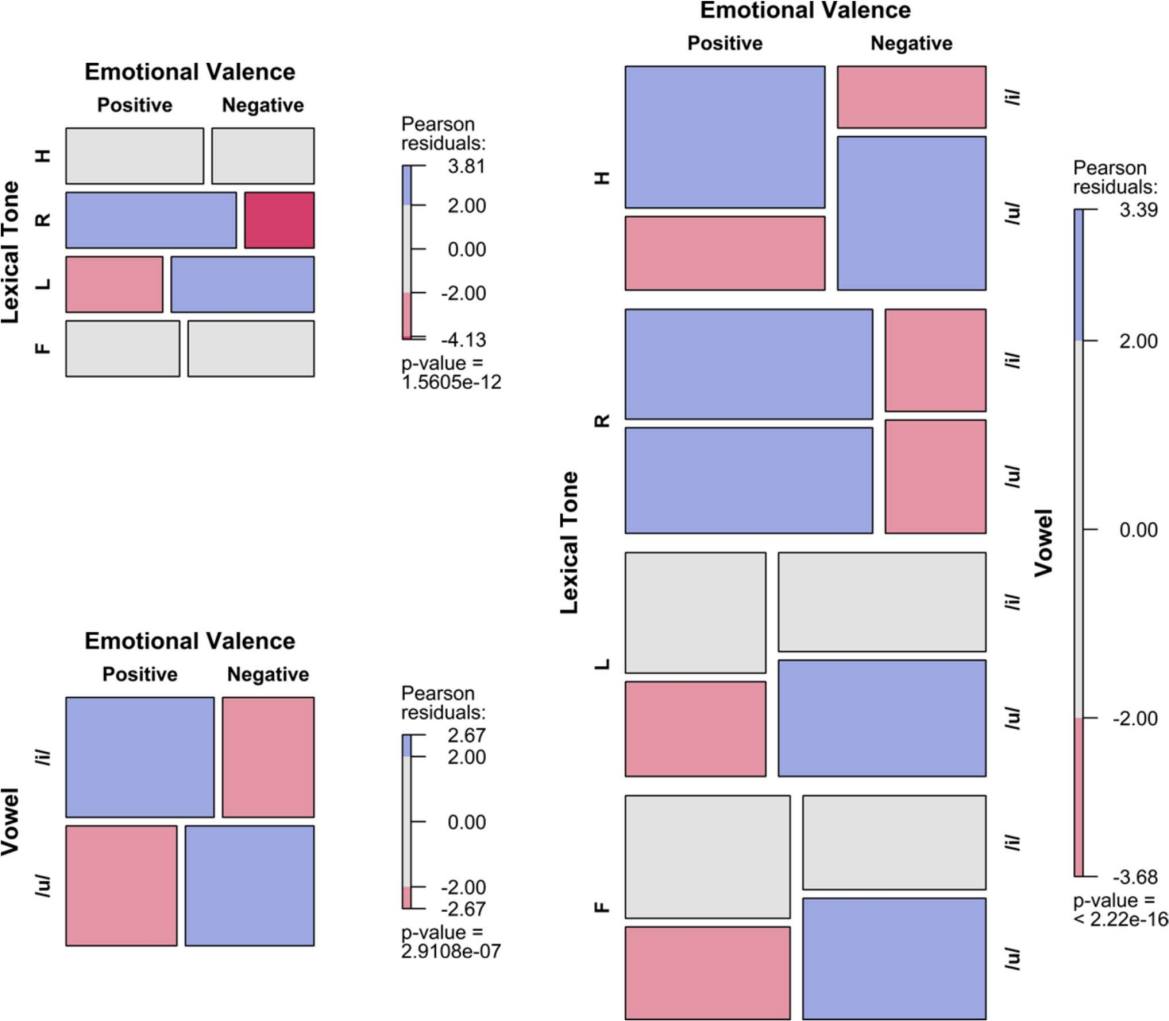
Valence frequency variations across SC tones, vowels, and their interactions in the V dataset. (Colour figure online)

**Fig. 4:**
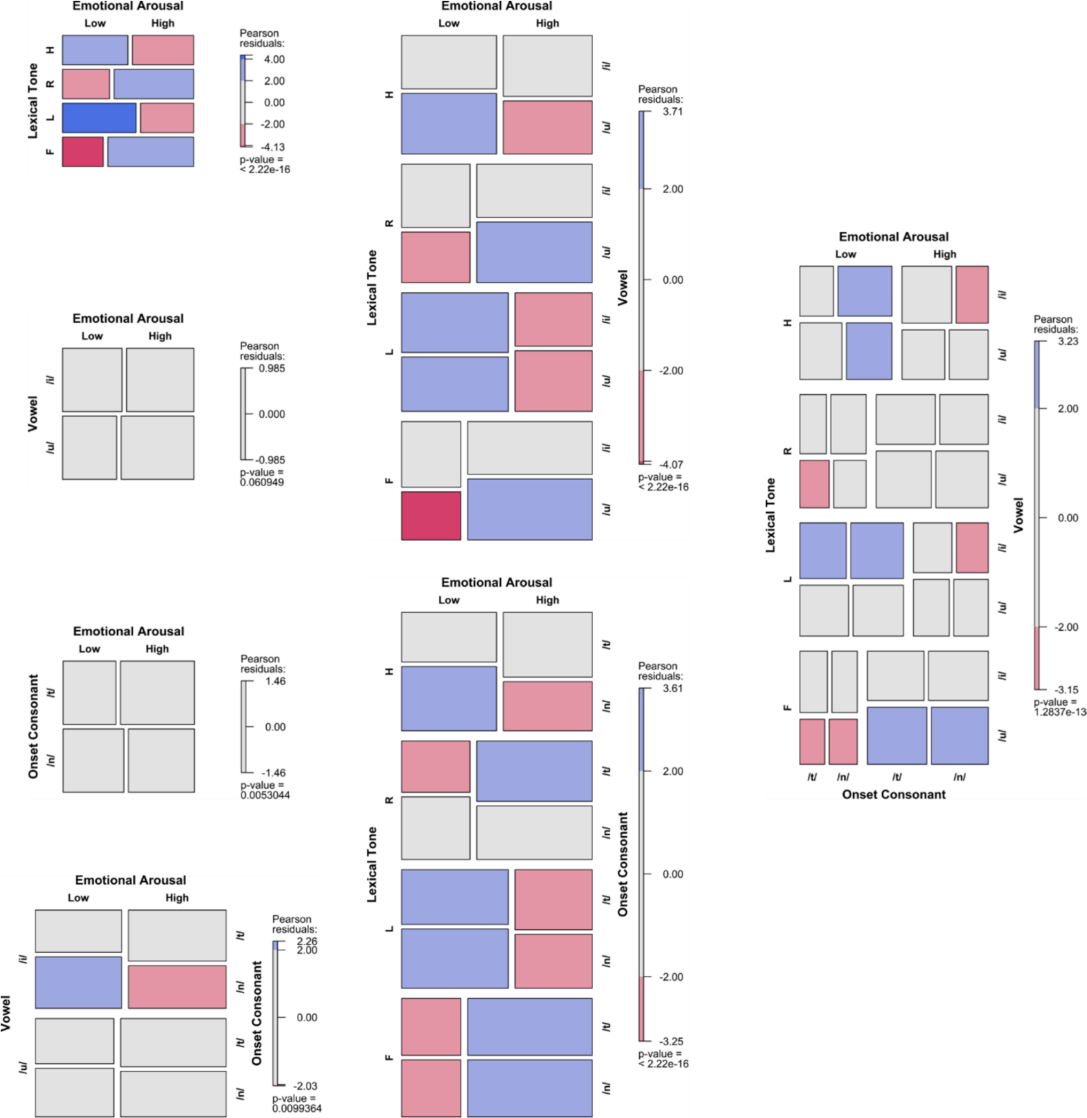
Arousal frequency variations across SC tones, vowels, onset consonants, and their interactions in the CVL dataset. (Colour figure online)

**Fig. 5:**
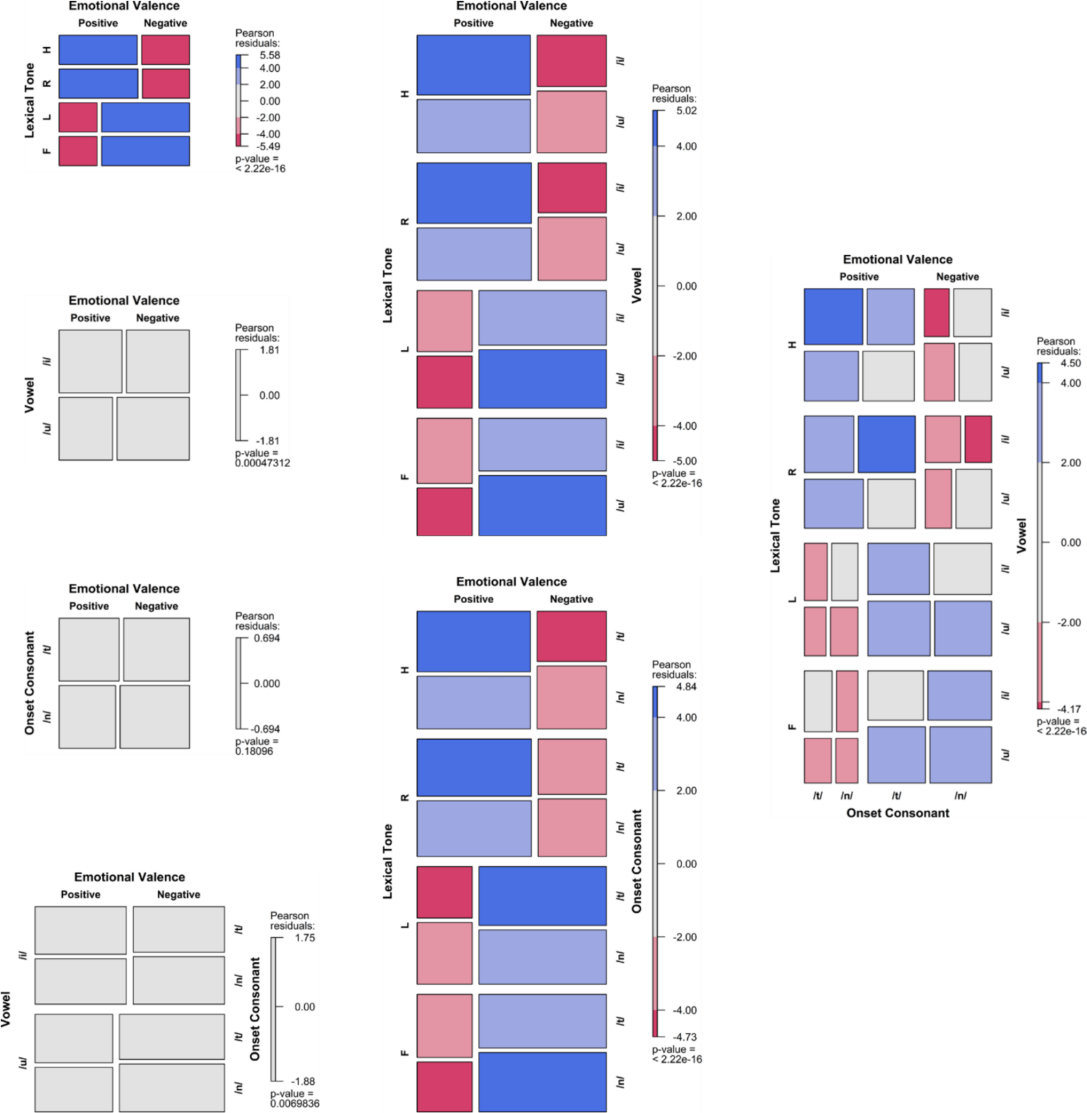
Valence frequency variations across SC tones, vowels, onset consonants, and their interactions in the CVL dataset. (Colour figure online)

Figures 2, 3, 4 and 5 present mosaic plots illustrating the frequency distribution of arousal or valence choices across various predictors, including tone, vowel, onset consonant, and their interactions. Residual ratios, ranging from 0.93 to 1.01, indicate no concerns regarding overdispersion across all logistic regression models.

### V dataset

#### Arousal

The random effect of the variable subject was negligible across all models (i.e., singular fit), indicating insignificant variability in arousal ratings attributed to individual differences, and it was thus excluded from all the alternative models. All alternative models significantly predicted the emotional arousal ratings of stimuli. Specifically, model (a) yielded χ^2^ = 164.14, *p* < .001, with an *R*^2^ of 7.13%; (b) χ^2^ = 4.26, *p* < .05, *R*^2^* =* 0.32%; and (c) χ^2^= 171.83, *p* < .001, *R*^2^* =* 12.85%.

Pairwise comparisons indicated that T4 was rated as significantly more arousing than T1 and T3 (*OR* = 3.20, 5.48), as was T2 compared with T1 and T3 (*OR* = 5.26, 9.09). However, no significant difference was found between /i/ and /u/ in arousal ratings (*p* = .12). The significant interaction between tone and vowel primarily reflected the main effect of tone, suggesting nonsignificant influence of single vowels on arousal ratings.

#### Valence

In contrast to arousal ratings, the random effect of the variable subject was significant, indicating some variability in valence ratings attributed to individual differences. The variance was 0.04 (±0.21) for model (a), 0.02 (±0.16) for model (b), and 0.08 (±0.27) for model (c). All alternative models significantly predicted the emotional valence ratings of the utterances. Specifically, model (a) yielded χ^2^ = 59.63, *p* < .001, with an *R*^2^ of 4.00%; for model (b) χ^2^ = 26.53, *p* < .001, *R*^2^* =* 1.80%; and for model (c) χ^2^ = 105.31, *p* < .001, *R*^2^* =* 7.07%.

Pairwise comparisons showed that T4, T1, and T3 were more likely to be rated negatively than T2 (*OR* = 2.76, 1.84, and 3.69, respectively). Moreover, T3 was twice as likely to be rated negatively as T1. Additionally, /u/ was rated more negatively than /i/ (*OR* = 1.89). The interaction effect between tone and vowel revealed that T2 consistently predicted positive valence regardless of vowel identity, while /i/ paired with T3 was rated as more negative (*OR* = 2.83) than /u/ paired with T2, suggesting a predominant effect of tone (over vowel) on valence ratings.

### CVL dataset

#### Arousal

The random effect of the subject was significant in models (a), (c), and (e) with a variance of 0.25 (±0.50), as well as in models (b), (d) and (f) with variance of 0.21 (±0.46). Across all alternative models, fixed effects significantly predicted the emotional arousal rating. Specifically, model (a) yielded χ^2^ = 91.33, *p* < .001, with an *R*^2^ of 3.46%; (b) χ^2^ = 3.69, *p* = .05, *R*^*2*^* =* 0.14%; (c) χ^2^ = 104.60, *p* < .001, *R*^2^ *=* 4.00%; (d) χ^2^ = 0.17, *p* < .01,* R*^2^* =* 0.31%; (e) χ^2^ = 104.46, *p* < .001, *R*^2^* =* 3.96%; (f) χ^2^ = 13.93, *p* < .01, *R*^2^ *=* 0.53%; and (g) χ^2^ = 94.64, *p* < .001, *R*^*2*^* =* 3.59%.

Pairwise multiple comparisons demonstrated that T4 was rated to be more arousing than T1 and T3 (*OR* = 2.35, 3.10), as was T2 compared to T1 and T3 (*OR* = 1.85, 2.44). Additionally, /t/ predicted higher arousal than /n/ (*OR* = 1.31). These effects of tone and consonant were consistent with their main effects in the interaction models. Furthermore, /n4/ and /n2/ predicted higher arousal than /t3/ (*OR* = 2.78 and 1.89) in model (e). In model (g), /nu4/ was more likely to be rated as higher arousal than /ti3/, /tu1/, and /tu3/ (*OR* = 3.23, 2.78 and 2.94, respectively). These interaction effects suggest a predominant effect of tone on the arousal rating over onset consonant. Again, the vowel effect on arousal rating was not observed.

#### Valence

The random effect of the subject was significant except for model (g), with variances of 0.17 (±0.41) for models (a), (c) and (e), and 0.10 (±0.32) for models (b) and (d). For model (f), it was 0.11 (±0.32). All alternative models significantly predicted the emotional valence ratings of the nonce words, except for model (d). Specifically, model (a) yielded χ^2^ = 235.78, *p* < .001, with an *R*^2^ of 7.93%; (b) χ^2^ = 12.54, *p* < .001, *R*^2^* =* 0.42%; (c) χ^2^ = 234.85, *p* < .001, *R*^2^* =* 7.90%; (d) χ^2^ = 1.84, *p* = .176,* R*^2^* =* 0.06%; (e) χ^2^ = 245.58, *p* < .001, *R*^2^* =* 8.26%; (f) χ^2^ = 14.49, *p* < .01, *R*^2^ = 0.49%; and (g) χ^2^ = 253.71, *p* < .001, *R*^2^* =* 8.53%.

Pairwise multiple comparisons revealed that T4 was rated to be more negative than T1 and T2 (*OR* = 3.99, 4.08), as was T3 compared to T1 and T2 (*OR* = 4, 4.08). Vowel /u/ was rated to be more negative than /i/ (*OR* = 1.37). Regarding the interaction effect of tone and vowel, /i4/ was rated to be more negative than /u1/ and /u2/ (*OR* = 2.75, 2.71), as was /i3/ compared to /u1/ and /u2/ (*OR* = 3.05, 3.00). These results suggest a predominant effect of tone on valence ratings over vowel, reconfirmed in model (g). No significant effect of consonants on valence ratings was observed.

## Discussion

The present study investigated how tone, vowel, consonant, and their interactions contribute to the ratings of emotional arousal and valence by native Standard Chinese speakers. Results showed significant tone–arousal, consonant–arousal, tone–valence, and vowel–valence associations, suggesting that despite their phonemic status in Standard Chinese, both segments and tones influence listeners’ ratings of the affective meanings. These findings extend previous research suggesting that pitch variation in lexical tones can shape emotional responses (Zheng et al., 2024, 2025), indicating that comparable affective patterns emerge even when these tonal pitch contours are realised on phonologically legal nonce words. Additionally, the study replicates previously reported phoneme–emotion correspondences, lending further support to the view that systematic form–meaning mappings may occur across languages (Svantesson, 2017). However, the absence of consonant–valence and vowel–arousal correspondences warrant further examination.

One novel discovery of this study is the predominant impact of lexical tone over consonants on arousal rating and over vowels on valence rating. Lexical–prosodic information has been considered a facilitator in interpreting iconic meanings across languages (Dingemanse et al., 2016; Perlman et al., 2015; Thompson, 2018). Our results highlight that, although lexical tone in a tonal language primarily serves to distinguish word meanings, speakers nevertheless seem to prioritise the pitch patterns of lexical tones when interpreting the overall affective meaning of a nonce word, which is particularly evident in cases when the tonal and segmental cues are not synergistic (e.g., /i4/ versus /u1/). This expands the view of prosody as a facilitating element, highlighting its critical role in shaping affective responses. It is worth noting that because the current design included only four segments, future research is needed to test whether this predominant tonal effect generalises to other phonemic contexts and stimulus sets.

Associations between phonemic segments and affective meanings likely arise from both articulatory and acoustic properties. Existing research suggests that articulatory gestures can evoke embodied emotional cues (e.g., smiling or frowning configurations), while acoustic features such as pitch, spectral energy, and formant structure influence affective perception. The consonant–arousal resemblance “/t/–high arousal, /n/–low arousal” may partially reflect these mechanisms. Specifically, the plosive /t/, involving a complete closure followed by an explosive release of airflow, produces a sudden burst with higher spectral energy and can consequently bias sensory and affective evaluations (Aryani et al., 2018; Winter & Perlman, 2021). In contrast, the nasal /n/ produces lower-frequency and continuous voicing, and can therefore be linked with softness and roundness (Berlin, 2006; Sakamoto & Watanabe, 2018). Given that arousal reflects physiological activation, we cautiously infer that acoustic energy and spectral dynamics may contribute to the observed consonant–arousal patterns.

Similarly, the “/i/–positive, /u/–negative” correspondence, consistent with findings in Japanese and German (Körner & Rummer, 2023), are also likely to stem from both articulatory and acoustic factors. Articulatorily, /i/ involves facial tension resembling a smile, whereas /u/ involves more rounded, closed configurations associated with negative affect (Körner & Rummer, 2023). Acoustically, /i/ typically shows higher pitch and second formant frequencies than /u/ (as discussed in Aryani et al., 2013, 2018; Knoeferle et al., 2017; Ohala, 1994; Perlman & Cain, 2014). While most of the existing studies did not directly test the causal link between second formant frequency and emotional valence, Auracher et al. (2020) reported small yet reliable formant differences that affect the perceived emotions of poetic speech. These converging findings motivate future replications to further test how articulatory and acoustic characteristics of speech sounds jointly shape emotional perception.

It is worth noting that in the vowel-only condition, participants’ judgments may have been partially influenced by associations with existing homophones or interjections. For instance, /u/ with T1 and /i/ with T2 may evoke the morphemes 污 (“dirty”) and 咦 (“surprise”), respectively. However, we did observe similar vowel-valence associations in the CVL nonce words, which suggests that these effects could not have been solely driven by conventionalised lexical forms. Additionally, the use of auditory presentation helped minimise potential confounds related to orthography or typography (Cuskley et al., 2017).

The influence of lexical tones on perceived emotional arousal and valence appears to be shaped by their pitch characteristics. Higher arousal ratings for T4- and T2-syllables, relative to T1- and T3-syllables, likely result from more dynamic pitch variations of the falling and rising tones. Although pitch height may contribute (e.g., T4/T2 versus T3), the minimal difference between T4/T2 and T1 suggests that pitch dynamics, rather than static height, play a more decisive role (Zheng et al., 2025). Prior studies using disyllabic words found T4 to elicit higher arousal ratings than T2 (Zheng et al., 2024, 2025), whereas the comparable arousal ratings for T4 and T2 in the present nonce-word context suggest that lexical semantics may modulate but do not fully determine tonal affect. Replication using acoustically controlled stimuli is needed to clarify these effects.

In terms of valence, the relatively higher positivity ratings for T1 and T2 (versus T4 and T3) may partly reflect their higher pitch levels (e.g., T1 > T4/T3; T2 > T3) and, in the case of T2, larger pitch ranges (T2 > T3). Although T4 exhibits both a greater pitch range and higher pitch level, its falling contour may contribute to a more negative perceived valence than T2. This pattern aligns with prior findings that rising contours (e.g., T2) tend to be associated with positive affect, whereas falling contours (e.g., T4) tend to convey less positive affect (Yap et al., 2014; Zheng et al., 2024). These associations, however, should be viewed as probabilistic tendencies rather than categorical one-to-one mappings (Morton, 1977; Perlman, 2024; Winter et al., 2021). Further research is needed to disentangle the relative contributions of pitch trajectory, overall height, and range in shaping affective valence.

The iconicity of pitch has been widely discussed within the *frequency code* framework (Gussenhoven, 2016; Hinton et al., 2006; Ohala, 1983, 1984, 1994), which posits that higher or rising pitch is associated with smaller body size and conveys social-affective meanings such as politeness, submission, and deference, signalling non-threat and a desire for goodwill or cooperation. In contrast, lower or falling pitch is associated with aggression, authority, and confidence. These social-affective cues are not only tied to the potency/dominance dimension in the Evaluation-Dominance-Activity model proposed by Osgood et al. (1957), but also carry valence implications. For instance, politeness and deference are generally evaluated positively, whereas aggression often has negative connotations, particularly in interpersonal or social-affective contexts. The tone–emotion associations observed in this study are broadly compatible with this hypothesis: the relatively more positive ratings of tones with high and rising pitch patterns (e.g., T1 and T2) reflect a possible link between pitch characteristics and emotional valence. Our results therefore provide another case where the frequency code may structure a sound-symbolic connection between pitch and affective interpretation. Again, it is important to note that the mapping between pitch characteristics and their possible interpretations is by no means straightforward and is most likely probabilistic and context-dependent. Furthermore, while our results suggest that certain sound–emotion associations may stem from universal perceptual mechanisms, cross-linguistic differences must also be acknowledged. Affective iconicity likely emerges from an interaction between general perceptual tendencies—rooted in acoustic and articulatory properties—and language-specific phonological patterns and cultural conventions. Future cross-linguistic studies should therefore consider these factors when interpreting affective iconicity.

A key limitation of this study is the restricted stimulus set. While we included all four lexical tones in Standard Chinese, only four segments (/i/, /u/, /t/, /n/) were included to explore tonal–segmental interactions. Expanding the phonemic inventory and systematically manipulating acoustic parameters (e.g., spectral energy, formant spacing, pitch range) would allow stronger tests of causality and generalizability.

Future studies could further investigate the systematicity of phoneme–meaning correspondences in SC, following approaches such as Schmidtke et al. (2014), to provide a comprehensive overview of sound-symbolic patterns. While our previous work has demonstrated affective iconicity in SC lexical tones (Zheng et al., 2024, 2025), these findings should not be taken as evidence for systematicity in lexical tone affective iconicity, particularly considering the well-accepted theory of tonogenesis, which posits that lexical tones historically emerged from segmental features (e.g., Michaud & Sands, 2020). Instead, it is the pitch variation patterns associated with the lexical tones that appear to convey affective meaning through iconic mechanisms. Further research is needed to evaluate the universality of these effects and identify potential cross-linguistic differences. It is also important to note that our study focused specifically on segmental and suprasegmental features, while other factors, such as syllabic structure, stress, morphological complexity, and affective form typicality, have been shown to influence sound-symbolic associations as well (e.g., De Zubicaray & Hinojosa, 2024; De Zubicaray et al., 2023; Schmidtke & Conrad, 2024). These dimensions warrant closer attention in future research.

In summary, the present findings provide evidence that consonants, vowels, and tones each contribute to affective interpretations of Standard Chinese vowels and nonce words, revealing multilayered sound–emotion correspondences at the sublexical level. Understanding how these cues interact can advance our knowledge of language evolution and the embodied foundations of emotional expression (e.g., Imai & Kita, 2014; Sidhu, 2025; Zheng et al., 2024). In lexical tone languages such as Standard Chinese, tones introduce a suprasegmental layer of affective meaning that complements segmental features, enabling emotional expression even in the absence of semantic content. These results suggest that affective iconicity likely functions as an automatic, embodied mechanism linking sound and emotion—one that contributes to the expressive richness of spoken language across diverse linguistic systems. Future research could further explore how tones and emotional prosody interact in Standard Chinese as well as other languages with typologically different tone systems.

## Supplementary Information

Below is the link to the electronic supplementary material.

Supplementary file1 (DOCX 170 KB)
