# Supplementary material for: The affective iconicity of segment and tone in Standard Chinese
Source: Psychon Bull Rev. 2026 Feb 17;33(3):67. doi: 10.3758/s13423-025-02849-5 (PMC12913264; doi:10.3758/s13423-025-02849-5)
Supplement: Supplementary file 1 — Supplementary file1 (DOCX 170 KB) [file 13423_2025_2849_MOESM1_ESM.docx]

**The Affective Iconicity of Segment and Tone in Standard Chinese**

**Supplementary Tables**

# **1 Arousal in Single Vowel Dataset**

**Table S1.1**

*Coefficients of the GLMM for Arousal of Tones in Vowels*

| Predictor | B (LogOdds) | Odds | SE B  (std. Error) | *Z* | *p* |
| --- | --- | --- | --- | --- | --- |
| Intercept (Tone F) | 0.7621401 | 2.1428571 | 0.1380131 | 5.522231 | 3.35E-08*** |
| H vs. F | -1.1641628 | 0.3121839 | 0.1904034 | -6.114193 | 9.70E-10*** |
| L vs. F | -1.7016876 | 0.1823755 | 0.1987475 | -8.562056 | 1.11E-17*** |
| R vs. F | 0.4853179 | 1.6246914 | 0.2070875 | 2.34354 | 1.91E-02* |

*Signif. codes (Apply to all tables): 0 ‘***’ 0.001 ‘**’ 0.01 ‘*’ 0.05 ‘.’ 0.1 ‘ ’ 1. Tone. Codes (Apply to all tables): H, Tone1; R, Tone 2; L, Tone 3; F, Tone 4.*

**Table S1.2**

*Pairwise Multiple Comparisons of the GLMM for Arousal among Tones in Vowels*

| Predictor | B (LogOdds) | Odds | SE B  (std. Error) | *Z* | *p* |
| --- | --- | --- | --- | --- | --- |
| **F-H** | **1.1641628** | **3.2032401** | **0.1904034** | **6.114193** | **9.70E-09***** |
| **F-L** | **1.7016876** | **5.4831933** | **0.1987475** | **8.562056** | **1.11E-16***** |
| F-R | -0.4853179 | 0.6155015 | 0.2070875 | -2.34354 | 1.91E-01^.^ |
| H-L | 0.5375248 | 1.7117647 | 0.1940588 | 2.769907 | 5.61E-02^.^ |
| **H-R** | **-1.6494807** | **0.1921497** | **0.2025919** | **-8.14189** | **3.89E-15***** |
| **L-R** | **-2.1870055** | **0.1122524** | **0.2104534** | **-10.391877** | **2.70E-24***** |

**Table S1.3**

*Coefficients of the GLMM for Arousal of Vowels*

| Predictor | B (LogOdds) | Odds | SE B  (std. Error) | *Z* | *p* |
| --- | --- | --- | --- | --- | --- |
| Intercept (Vowel /i/) | 0.2744368 | 1.3157895 | 0.0917647 | 2.990658 | 0.002783771** |
| /u/ vs. /i/ | -0.2661723 | 0.7663071 | 0.1291718 | -2.060606 | 0.0393406* |

**Table S1.4**

*Pairwise Multiple Comparisons of the GLMM for Arousal between Vowels*

| Predictor | B (LogOdds) | Odds | SE B  (std. Error) | *Z* | *p* |
| --- | --- | --- | --- | --- | --- |
| /i/-/u/ | 0.2661723 | 1.30496 | 0.1291718 | 2.060606 | 0.1180218 |

**Table S1.5**

*Coefficients of the GLMM for Arousal of Tones by Vowels*

| Predictor | B (LogOdds) | Odds | SE B  (std. Error) | *Z* | *p* |
| --- | --- | --- | --- | --- | --- |
| Intercept (ToneF:Vowel/i/) | 0.9808293 | 2.6666667 | 0.204124 | 4.8050661 | 1.55E-06*** |
| H vs. F | -1.0966611 | 0.3339844 | 0.2735607 | -4.0088398 | 6.10E-05*** |
| L vs. F | -1.9203768 | 0.1465517 | 0.2873544 | -6.6829556 | 2.34E-11*** |
| R vs. F | 0.3646431 | 1.44 | 0.3034522 | 1.2016493 | 2.29E-01 |
| /u/ vs. /i/ | -0.4212135 | 0.65625 | 0.2781742 | -1.5142075 | 1.30E-01 |
| H:/u/ vs. F:/i/ | -0.1685244 | 0.8449106 | 0.3845717 | -0.4382132 | 6.61E-01 |
| L:/u/ vs. F:/i/ | 0.4212135 | 1.5238095 | 0.3989897 | 1.0557 | 2.91E-01 |
| R:/u/ vs. F:/i/ | 0.2302338 | 1.2588944 | 0.4161127 | 0.5532969 | 5.80E-01 |

**Table S1.6**

*Pairwise Multiple Comparisons of the GLMM for Arousal among Tones and Vowels*

| Predictor | B (LogOdds) | Odds | SE B  (std. Error) | *Z* | *p* |
| --- | --- | --- | --- | --- | --- |
| **F/i/-H/i/** | **1.10E+00** | **2.994152** | **0.2735607** | **4.01E+00** | **2.20E-03**** |
| **F/i/-L/i/** | **1.92E+00** | **6.8235294** | **0.2873544** | **6.68E+00** | **8.43E-10***** |
| F/i/-R/i/ | -3.65E-01 | 0.6944444 | 0.3034522 | -1.20E+00 | 1.00E+00 |
| F/i/-F/u/ | 4.21E-01 | 1.5238095 | 0.2781742 | 1.51E+00 | 1.00E+00 |
| **F/i/-H/u/** | **1.69E+00** | **5.4** | **0.2810912** | **6.00E+00** | **7.13E-08***** |
| **F/i/-L/u/** | **1.92E+00** | **6.8235294** | **0.2873544** | **6.68E+00** | **8.43E-10***** |
| F/i/-R/u/ | -1.74E-01 | 0.8405797 | 0.2949896 | -5.89E-01 | 1.00E+00 |
| H/i/-L/i/ | 8.24E-01 | 2.2789522 | 0.2721669 | 3.03E+00 | 8.91E-02 |
| **H/i/-R/i/** | **-1.46E+00** | **0.2319336** | **0.2891116** | **-5.05E+00** | **1.55E-05***** |
| H/i/-F/u/ | -6.75E-01 | 0.5089286 | 0.262456 | -2.57E+00 | 3.62E-01 |
| H/i/-H/u/ | 5.90E-01 | 1.8035156 | 0.2655457 | 2.22E+00 | 9.49E-01 |
| H/i/-L/u/ | 8.24E-01 | 2.2789522 | 0.2721669 | 3.03E+00 | 8.91E-02 |
| **H/i/-R/u/** | **-1.27E+00** | **0.2807405** | **0.2802162** | **-4.53E+00** | **2.09E-04***** |
| **L/i/-R/i/** | **-2.29E+00** | **0.101772** | **0.3021963** | **-7.56E+00** | **1.44E-12***** |
| **L/i/-F/u/** | **-1.50E+00** | **0.2233169** | **0.2768036** | **-5.42E+00** | **2.19E-06***** |
| L/i/-H/u/ | -2.34E-01 | 0.7913793 | 0.2797349 | -8.36E-01 | 1.00E+00 |
| L/i/-L/u/ | -8.16E-15 | 1 | 0.2860278 | -2.85E-14 | 1.00E+00 |
| **L/i/-R/u/** | **-2.09E+00** | **0.1231884** | **0.2936975** | **-7.13E+00** | **3.62E-11***** |
| R/i/-F/u/ | 7.86E-01 | 2.1942857 | 0.2934807 | 2.68E+00 | 2.67E-01 |
| **R/i/-H/u/** | **2.05E+00** | **7.776** | **0.296247** | **6.92E+00** | **1.59E-10***** |
| **R/i/-L/u/** | **2.29E+00** | **9.8258824** | **0.3021963** | **7.56E+00** | **1.44E-12***** |
| R/i/-R/u/ | 1.91E-01 | 1.2104348 | 0.3094655 | 6.17E-01 | 1.00E+00 |
| **F/u/-H/u/** | **1.27E+00** | **3.54375** | **0.270296** | **4.68E+00** | **1.03E-04***** |
| **F/u/-L/u/** | **1.50E+00** | **4.4779412** | **0.2768036** | **5.42E+00** | **2.19E-06***** |
| F/u/-R/u/ | -5.95E-01 | 0.5516304 | 0.2847218 | -2.09E+00 | 1.00E+00 |
| H/u/-L/u/ | 2.34E-01 | 1.2636166 | 0.2797349 | 8.36E-01 | 1.00E+00 |
| **H/u/-R/u/** | **-1.86E+00** | **0.1556629** | **0.2875724** | **-6.47E+00** | **3.57E-09***** |
| **L/u/-R/u/** | **-2.09E+00** | **0.1231884** | **0.2936975** | **-7.13E+00** | **3.62E-11***** |

**Table S1.7**

*Post-hoc Power of the Arousal Models for Tones and Vowels*

| Model | Predictor | Effect size/Odds | *Power* |
| --- | --- | --- | --- |
| Arousal ~ Tone | Intercept (Tone F) | 2.1428571 | 1 |
|  | H vs. F | 0.3121839 | 0.9999995 |
|  | L vs. F | 0.1823755 | 0.9799296 |
|  | R vs. F | 1.6246914 | 1 |
| Arousal ~ Vowel | Intercept (Vowel /i/) | 1.3157895 | 1 |
|  | /u/ vs. /i/ | 0.7663071 | 1 |
| Arousal ~ Tone * Vowel | Intercept (ToneF:Vowel/i/) | 2.6666667 | 1 |
|  | H vs. F | 0.3339844 | 1 |
|  | L vs. F | 0.1465517 | 0.8969163 |
|  | R vs. F | 1.44 | 1 |
|  | /u/ vs. /i/ | 0.65625 | 1 |
|  | H:/u/ vs. F:/i/ | 0.8449106 | 1 |
|  | L:/u/ vs. F:/i/ | 1.5238095 | 1 |
|  | R:/u/ vs. F:/i/ | 1.2588944 | 1 |

# **2 Valence in Single Vowel Dataset**

**Table S2.1**

*Coefficients of the GLMM for Valence of Tones in Vowels*

| Predictor | B (LogOdds) | Odds | SE B  (std. Error) | *Z* | *p* |
| --- | --- | --- | --- | --- | --- |
| Intercept (Tone F) | 0.1049366 | 1.1106401 | 1.24E-01 | 0.8471973 | 3.97E-01 |
| H vs. F | -0.4066702 | 0.6658638 | 1.74E-01 | -2.3332313 | 1.96E-02* |
| L vs. F | 0.2893411 | 1.3355473 | 1.75E-01 | 1.6543197 | 9.81E-02 |
| R vs. F | -1.0149324 | 0.3624269 | 1.83E-01 | -5.5448869 | 2.94E-08*** |

**Table S2.2**

*Pairwise Multiple Comparisons of the GLMM for Valence among Tones in Vowels*

| Predictor | B (LogOdds) | Odds | SE B  (std. Error) | *Z* | *p* |
| --- | --- | --- | --- | --- | --- |
| F-H | 0.4066702 | 1.50E+00 | 1.74E-01 | 2.333231 | 1.96E-01 |
| F-L | -0.2893411 | 7.49E-01 | 1.75E-01 | -1.65432 | 9.81E-01 |
| **F-R** | **1.0149324** | **2.76E+00** | **1.83E-01** | **5.544887** | **2.94E-07***** |
| **H-L** | **-0.6960113** | **4.99E-01** | **1.76E-01** | **-3.952309** | **7.74E-04***** |
| **H-R** | **0.6082622** | **1.84E+00** | **0.1832943** | **3.318501** | **9.05E-03**** |
| **L-R** | **1.3042735** | **3.69E+00** | **0.185039** | **7.04864** | **1.81E-11***** |

**Table S2.3**

*Coefficients of the GLMM for Valence of Vowels*

| Predictor | B (LogOdds) | Odds | SE B  (std. Error) | *Z* | *p* |
| --- | --- | --- | --- | --- | --- |
| Intercept (Vowel /i/) | -0.4861487 | 0.6149903 | 0.0902039 | -5.389443 | 7.07E-08*** |
| /u/ vs. /i/ | 0.6354661 | 1.8879019 | 0.1244881 | 5.104633 | 3.31E-07*** |

**Table S2.4**

*Pairwise Multiple Comparisons of the GLMM for Valence between Vowels*

| Predictor | B (LogOdds) | Odds | SE B  (std. Error) | *Z* | *p* |
| --- | --- | --- | --- | --- | --- |
| **/i/-/u/** | **-0.6354661** | **5.30E-01** | **0.1244881** | **-5.104633** | **9.94E-07***** |

**Table S2.5**

*Coefficients of the GLMM for Valence of Tones by Vowels*

| Predictor | B (LogOdds) | Odds | SE B  (std. Error) | *Z* | *p* |
| --- | --- | --- | --- | --- | --- |
| Intercept (ToneF:Vowel/i/) | -0.166511 | 0.8466135 | 0.1759518 | -0.9463441 | 0.343973095 |
| H vs. F | -0.9812747 | 0.374833 | 0.2671109 | -3.6736604 | 0.000239101*** |
| L vs. F | 0.3634091 | 1.4382241 | 2.47E-01 | 1.4724271 | 0.140905585 |
| R vs. F | -0.8247082 | 0.4383629 | 2.62E-01 | -3.1533472 | 0.001614097** |
| /u/ vs. /i/ | 0.5482749 | 1.7302656 | 2.49E-01 | 2.2063129 | 0.0273621* |
| H:/u/ vs. F:/i/ | 1.0441712 | 2.8410428 | 3.66E-01 | 2.8496888 | 0.004376203** |
| L:/u/ vs. F:/i/ | -0.1395651 | 0.8697364 | 3.54E-01 | -0.3947396 | 0.693035068 |
| R:/u/ vs. F:/i/ | -0.4016648 | 0.669205 | 3.68E-01 | -1.0928932 | 0.274440691 |

**Table S2.6**

*Pairwise Multiple Comparisons of the GLMM for Valence among Tones and Vowels*

| Predictor | B (LogOdds) | Odds | SE B  (std. Error) | *Z* | *p* |
| --- | --- | --- | --- | --- | --- |
| **F/i/-H/i/** | **0.98127475** | **2.6678549** | **0.2671109** | **3.6736604** | **8.61E-03**** |
| F/i/-L/i/ | -0.36340907 | 0.695302 | 0.2468096 | -1.4724271 | 1.00E+00 |
| F/i/-R/i/ | 0.8247082 | 2.281215 | 0.2615342 | 3.1533472 | 5.81E-02 |
| F/i/-F/u/ | -0.54827494 | 0.5779459 | 0.2485028 | -2.2063129 | 9.85E-01 |
| F/i/-H/u/ | -0.61117136 | 0.5427148 | 0.2493268 | -2.4512867 | 5.12E-01 |
| F/i/-L/u/ | -0.77211895 | 0.462033 | 0.2520058 | -3.0638938 | 7.87E-02 |
| F/i/-R/u/ | 0.67809807 | 1.9701271 | 0.2571645 | 2.6368261 | 3.01E-01 |
| **H/i/-L/i/** | **-1.34468381** | **0.2606221** | **0.267836** | **-5.02055** | **1.85E-05***** |
| H/i/-R/i/ | -0.15656654 | 0.8550746 | 0.2800826 | -0.5590013 | 1.00E+00 |
| **H/i/-F/u/** | **-1.52954969** | **0.2166332** | **0.2696334** | **-5.6727021** | **5.06E-07***** |
| **H/i/-H/u/** | **-1.59244611** | **0.2034274** | **0.2704677** | **-5.8877493** | **1.41E-07***** |
| **H/i/-L/u/** | **-1.75339369** | **0.1731852** | **0.2731323** | **-6.419577** | **4.92E-09***** |
| H/i/-R/u/ | -0.30317668 | 0.7384686 | 0.2761545 | -1.0978515 | 1.00E+00 |
| **L/i/-R/i/** | **1.18811727** | **3.2808983** | **0.2622084** | **4.5311946** | **2.11E-04***** |
| L/i/-F/u/ | -0.18486588 | 0.8312158 | 0.2484521 | -0.7440705 | 1.00E+00 |
| L/i/-H/u/ | -0.2477623 | 0.7805455 | 0.2492403 | -0.9940701 | 1.00E+00 |
| L/i/-L/u/ | -0.40870988 | 0.664507 | 0.2518364 | -1.6229182 | 1.00E+00 |
| **L/i/-R/u/** | **1.04150714** | **2.8334842** | **0.2577815** | **4.0402708** | **1.92E-03**** |
| **R/i/-F/u/** | **-1.37298315** | **0.2533501** | **0.2640108** | **-5.2004817** | **7.16E-06***** |
| **R/i/-H/u/** | **-1.43587957** | **0.237906** | **0.2648514** | **-5.4214529** | **2.13E-06***** |
| **R/i/-L/u/** | **-1.59682715** | **0.2025381** | **0.267547** | **-5.9683991** | **8.63E-08***** |
| R/i/-R/u/ | -0.14661013 | 0.8636306 | 0.2708748 | -0.5412469 | 1.00E+00 |
| F/u/-H/u/ | -0.06289642 | 0.9390407 | 0.2507427 | -0.2508405 | 1.00E+00 |
| F/u/-L/u/ | -0.223844 | 0.7994398 | 0.2532815 | -0.8837755 | 1.00E+00 |
| **F/u/-R/u/** | **1.22637301** | **3.4088433** | **0.2595813** | **4.724428** | **8.31E-05***** |
| H/u/-L/u/ | -0.16094758 | 0.8513367 | 0.2540155 | -0.6336131 | 1.00E+00 |
| **H/u/-R/u/** | **1.28926943** | **3.6301335** | **0.2604281** | **4.9505767** | **2.66E-05***** |
| **L/u/-R/u/** | **1.45021702** | **4.2640398** | **0.2631397** | **5.5112048** | **1.28E-06***** |

**Table S2.7**

*Post-hoc Power of the Valence Models for Tones and Vowels*

| Model | Predictor | Effect size/Odds | *Power* |
| --- | --- | --- | --- |
| Valence ~ Tone | Intercept (Tone F) | 1.1106401 | 1 |
|  | H vs. F | 0.6658638 | 1 |
|  | L vs. F | 1.3355473 | 1 |
|  | R vs. F | 0.3624269 | 1 |
| Valence ~ Vowel | Intercept (Vowel /i/) | 0.6149903 | 1 |
|  | /u/ vs. /i/ | 1.8879019 | 1 |
| Valence ~ Tone * Vowel | Intercept (ToneF:Vowel/i/) | 0.8466135 | 1 |
|  | H vs. F | 0.374833 | 1 |
|  | L vs. F | 1.4382241 | 1 |
|  | R vs. F | 0.4383629 | 1 |
|  | /u/ vs. /i/ | 1.7302656 | 1 |
|  | H:/u/ vs. F:/i/ | 2.8410428 | 1 |
|  | L:/u/ vs. F:/i/ | 0.8697364 | 1 |
|  | R:/u/ vs. F:/i/ | 0.669205 | 1 |

The odds ratio is often used as a measure of effect size in logistic regression models because it provides a meaningful and interpretable summary of the relationship between a predictor variable and the outcome. The odds ratio quantifies how the odds of an event occurring (e.g., high arousal and negative valence) change with a one-unit increase in the predictor variable. An odds ratio of 1 suggests no change in the odds, a value greater than 1 indicates an increase in the odds, and a value less than 1 indicates a decrease in the odds.

The R-squared coefficient measures the proportion of the variance in the dependent variable that is explained by the independent variables in the model. R-squared values range from 0 to 1, where 0 indicates that the model does not explain any of the variability, and 1 indicates that the model explains all the variability in the outcome. Higher R-squared values indicate a better fit of the model to the data.

# **3 Arousal in CVL Nonce Word Dataset**

**Table S3.1**

*Coefficients of the GLMM for Arousal of Tones in Nonce Words*

| Predictor | B (LogOdds) | Odds | SE B  (std. Error) | *Z* | *p* |
| --- | --- | --- | --- | --- | --- |
| Intercept (Tone F) | 0.7851652 | 2.1927691 | 0.1099261 | 7.142666 | 9.15E-13*** |
| H vs. F | -0.8557741 | 0.4249541 | 0.1372599 | -6.2347 | 4.53E-10*** |
| L vs. F | -1.1300223 | 0.323026 | 0.1384111 | -8.164246 | 3.23E-16*** |
| R vs. F | -0.2310059 | 0.7937348 | 0.1388583 | -1.663609 | 9.62E-02^.^ |

**Table S3.2**

*Pairwise Multiple Comparisons of the GLMM for Arousal among Tones in Nonce Words*

| Predictor | B (LogOdds) | Odds | SE B  (std. Error) | *Z* | *p* |
| --- | --- | --- | --- | --- | --- |
| **F-H** | 0.8557741 | 2.35E+00 | 0.1372599 | 6.2347 | 4.53E-09*** |
| **F-L** | 1.1300223 | 3.10E+00 | 0.1384111 | 8.164246 | 3.23E-15*** |
| F-R | 0.2310059 | 1.26E+00 | 0.1388583 | 1.663609 | 9.62E-01 |
| H-L | 0.2742483 | 1.32E+00 | 0.1332303 | 2.058452 | 3.95E-01 |
| **H-R** | -0.6247682 | 0.5353855 | 0.134776 | -4.635606 | 3.56E-05*** |
| **L-R** | -0.8990165 | 0.4069697 | 0.1358641 | -6.617028 | 3.66E-10*** |

**Table S3.3**

*Coefficients of the GLMM for Arousal of Vowels in Nonce Words*

| Predictor | B (LogOdds) | Odds | SE B  (std. Error) | *Z* | *p* |
| --- | --- | --- | --- | --- | --- |
| Intercept (Vowel /i/) | 0.1300583 | 1.138895 | 0.07801458 | 1.667102 | 0.09549411^.^ |
| /u/ vs. /i/ | 0.1800547 | 1.197283 | 0.09376852 | 1.920204 | 0.05483216^.^ |

**Table S3.4**

*Pairwise Multiple Comparisons of the GLMM for Arousal between Vowels in Nonce Words*

| Predictor | B (LogOdds) | Odds | SE B  (std. Error) | *Z* | *p* |
| --- | --- | --- | --- | --- | --- |
| /i/-/u/ | -0.1800547 | 0.8352245 | 0.09376852 | -1.920204 | 0.1644965 |

**Table S3.5**

*Coefficients of the GLMM for Arousal of Tones by Vowels in Nonce Words*

| Predictor | B (LogOdds) | Odds | SE B  (std. Error) | *Z* | *p* |
| --- | --- | --- | --- | --- | --- |
| Intercept (ToneF:Vowel/i/) | 0.5548229 | 1.7416325 | 0.1442896 | 3.8452047 | 1.20E-04*** |
| H vs. F | -0.4852152 | 0.6155647 | 0.1904494 | -2.5477388 | 1.08E-02* |
| L vs. F | -1.0180783 | 0.3612886 | 0.1929255 | -5.2770536 | 1.31E-07*** |
| R vs. F | -0.1835297 | 0.8323272 | 0.1916333 | -0.9577128 | 3.38E-01 |
| /u/ vs. /i/ | 0.4812096 | 1.6180304 | 0.2013696 | 2.3896837 | 1.69E-02* |
| H:/u/ vs. F:/i/ | -0.7624135 | 0.4665391 | 0.2754125 | -2.76826 | 5.64E-03** |
| L:/u/ vs. F:/i/ | -0.2472857 | 0.7809175 | 0.2767304 | -0.8935981 | 3.72E-01 |
| R:/u/ vs. F:/i/ | -0.1060305 | 0.8993972 | 0.2796655 | -0.3791333 | 7.05E-01 |

**Table S3.6**

*Pairwise Multiple Comparisons of the GLMM for Arousal among Tones and Vowels in Nonce Words*

| Predictor | B (LogOdds) | Odds | SE B  (std. Error) | *Z* | *p* |
| --- | --- | --- | --- | --- | --- |
| F/i/-H/i/ | 0.48521522 | 1.6245246 | 0.1904494 | 2.54773876 | 3.90E-01 |
| **F/i/-L/i/** | **1.01807825** | **2.7678705** | **0.1929255** | **5.27705357** | **4.73E-06***** |
| F/i/-R/i/ | 0.18352966 | 1.2014506 | 0.1916333 | 0.95771278 | 1.00E+00 |
| F/i/-F/u/ | -0.48120962 | 0.6180354 | 0.2013696 | -2.38968366 | 6.07E-01 |
| **F/i/-H/u/** | **0.76641914** | **2.1520463** | **0.1910202** | **4.01224201** | **2.17E-03**** |
| **F/i/-L/u/** | **0.78415435** | **2.1905537** | **0.1911109** | **4.10313694** | **1.47E-03**** |
| F/i/-R/u/ | -0.19164947 | 0.8255962 | 0.195836 | -0.97862202 | 1.00E+00 |
| H/i/-L/i/ | 0.53286303 | 1.7038034 | 0.1895552 | 2.81112345 | 1.78E-01 |
| H/i/-R/i/ | -0.30168557 | 0.7395706 | 0.1886596 | -1.59910037 | 1.00E+00 |
| **H/i/-F/u/** | **-0.96642484** | **0.3804407** | **0.198832** | **-4.86051053** | **4.22E-05***** |
| H/i/-H/u/ | 0.28120391 | 1.3247237 | 0.1877487 | 1.49776746 | 1.00E+00 |
| H/i/-L/u/ | 0.29893912 | 1.3484275 | 0.1878303 | 1.59153834 | 1.00E+00 |
| **H/i/-R/u/** | **-0.67686469** | **0.5082079** | **0.1931099** | **-3.50507447** | **1.64E-02*** |
| **L/i/-R/i/** | **-0.8345486** | **0.4340704** | **0.1910612** | **-4.36796585** | **4.51E-04***** |
| **L/i/-F/u/** | **-1.49928787** | **0.2232891** | **0.2014319** | **-7.44315139** | **3.54E-12***** |
| L/i/-H/u/ | -0.25165912 | 0.7775097 | 0.1898387 | -1.32564675 | 1.00E+00 |
| L/i/-L/u/ | -0.23392391 | 0.791422 | 0.1899115 | -1.23175202 | 1.00E+00 |
| **L/i/-R/u/** | **-1.20972772** | **0.2982785** | **0.1956481** | **-6.18318213** | **2.26E-08***** |
| **R/i/-F/u/** | **-0.66473928** | **0.5144076** | **0.1998368** | **-3.32641033** | **3.17E-02*** |
| R/i/-H/u/ | 0.58288948 | 1.7912066 | 0.1891901 | 3.08097172 | 7.43E-02^.^ |
| R/i/-L/u/ | 0.60062469 | 1.8232574 | 0.1892779 | 3.17324231 | 5.43E-02^.^ |
| R/i/-R/u/ | -0.37517913 | 0.6871662 | 0.1942234 | -1.93168836 | 1.00E+00 |
| **F/u/-H/u/** | **1.24762875** | **3.4820763** | **0.1995111** | **6.25343079** | **1.45E-08***** |
| **F/u/-L/u/** | **1.26536397** | **3.5443825** | **0.1996034** | **6.33939003** | **8.30E-09***** |
| F/u/-R/u/ | 0.28956015 | 1.3358398 | 0.2037242 | 1.4213343 | 1.00E+00 |
| H/u/-L/u/ | 0.01773521 | 1.0178934 | 0.1881833 | 0.09424435 | 1.00E+00 |
| **H/u/-R/u/** | **-0.9580686** | **0.3836331** | **0.1937297** | **-4.94538745** | **2.74E-05***** |
| **L/u/-R/u/** | **-0.97580381** | **0.3768893** | **0.1938221** | **-5.0345328** | **1.72E-05***** |

**Table S3.7**

*Coefficients of the GLMM for Arousal of Consonants in Nonce Words*

| Predictor | B (LogOdds) | Odds | SE B  (std. Error) | *Z* | *p* |
| --- | --- | --- | --- | --- | --- |
| Intercept (Consonant /t/) | 0.3547256 | 1.4257893 | 0.07888079 | 4.496983 | 6.89E-06 |
| /n/ vs. /t/ | -0.2681884 | 0.7647636 | 0.0939257 | -2.855326 | 4.30E-03 |

**Table S3.8**

*Pairwise Multiple Comparisons of the GLMM for Arousal between Consonants in Nonce Words*

| Predictor | B (LogOdds) | Odds | SE B  (std. Error) | *Z* | *p* |
| --- | --- | --- | --- | --- | --- |
| **/t/-/n/** | **0.2681884** | **1.307594** | **0.0939257** | **2.855326** | **0.01289782*** |

**Table S3.9**

*Coefficients of the GLMM for Arousal of Tones by Consonants in Nonce Words*

| Predictor | B (LogOdds) | Odds | SE B  (std. Error) | *Z* | *p* |
| --- | --- | --- | --- | --- | --- |
| Intercept (ToneF:Consonant/t/) | 7.86E-01 | 2.1949277 | 0.1486539 | 5.2884526 | 1.23E-07*** |
| H vs. F | -5.76E-01 | 0.5623177 | 0.1940389 | -2.966870714 | 3.01E-03** |
| L vs. F | -1.03E+00 | 0.3558824 | 0.194575 | -5.309801563 | 1.10E-07*** |
| R vs. F | -5.93E-02 | 0.9424345 | 0.1986204 | -0.298503299 | 7.65E-01 |
| /n/ vs. /t/ | 8.32E-06 | 1.0000083 | 0.1995186 | 4.16946E-05 | 1.00E+00 |
| H:/n/ vs. F:/t/ | -5.65E-01 | 0.5684703 | 0.2748645 | -2.0548531 | 3.99E-02* |
| L:/n/ vs. F:/t/ | -1.98E-01 | 0.8204235 | 0.2753749 | -0.718782238 | 4.72E-01 |
| R:/n/ vs. F:/t/ | -3.38E-01 | 0.7135502 | 0.2783065 | -1.212700952 | 2.25E-01 |

**Table S3.10**

*Pairwise Multiple Comparisons of the GLMM for Arousal among Tones and Consonants in Nonce Words*

| Predictor | B (LogOdds) | Odds | SE B  (std. Error) | *Z* | *p* |
| --- | --- | --- | --- | --- | --- |
| F/t/-H/t/ | 5.76E-01 | 1.7783542 | 0.1940389 | 2.966870714 | 1.08E-01 |
| **F/t/-L/t/** | **1.03E+00** | **2.8099166** | **0.194575** | **5.309801563** | **3.95E-06***** |
| F/t/-R/t/ | 5.93E-02 | 1.0610817 | 0.1986204 | 0.298503299 | 1.00E+00 |
| F/t/-F/n/ | -8.32E-06 | 0.9999917 | 0.1995186 | -4.16946E-05 | 1.00E+00 |
| **F/t/-H/n/** | **1.14E+00** | **3.128289** | **0.1953212** | **5.839029889** | **1.89E-07***** |
| **F/t/-L/n/** | **1.23E+00** | **3.4249302** | **0.1961305** | **6.276846979** | **1.24E-08***** |
| F/t/-R/n/ | 3.97E-01 | 1.4870333 | 0.1949864 | 2.034926278 | 1.00E+00 |
| H/t/-L/t/ | 4.57E-01 | 1.5800658 | 0.1883611 | 2.428667771 | 5.46E-01 |
| H/t/-R/t/ | -5.16E-01 | 0.596665 | 0.1930854 | -2.674462383 | 2.69E-01 |
| H/t/-F/n/ | -5.76E-01 | 0.562313 | 0.1940466 | -2.966795912 | 1.08E-01 |
| H/t/-H/n/ | 5.65E-01 | 1.7590921 | 0.1890627 | 2.987357508 | 1.01E-01 |
| **H/t/-L/n/** | **6.55E-01** | **1.9258987** | **0.1898448** | **3.452256029** | **2.00E-02*** |
| H/t/-R/n/ | -1.79E-01 | 0.8361851 | 0.1891471 | -0.945852936 | 1.00E+00 |
| **L/t/-R/t/** | **-9.74E-01** | **0.3776203** | **0.1936031** | **-5.030219926** | **1.76E-05***** |
| **L/t/-F/n/** | **-1.03E+00** | **0.3558795** | **0.1945902** | **-5.309431799** | **3.96E-06***** |
| L/t/-H/n/ | 1.07E-01 | 1.1133031 | 0.1890799 | 0.567651021 | 1.00E+00 |
| L/t/-L/n/ | 1.98E-01 | 1.2188725 | 0.1898172 | 1.042720544 | 1.00E+00 |
| **L/t/-R/n/** | **-6.36E-01** | **0.529209** | **0.1895196** | **-3.357814588** | **2.83E-02*** |
| R/t/-F/n/ | -5.93E-02 | 0.9424267 | 0.1986325 | -0.298526957 | 1.00E+00 |
| **R/t/-H/n/** | **1.08E+00** | **2.9482075** | **0.1943394** | **5.563449085** | **9.52E-07***** |
| **R/t/-L/n/** | **1.17E+00** | **3.2277724** | **0.1951445** | **6.004742603** | **6.90E-08***** |
| R/t/-R/n/ | 3.37E-01 | 1.4014315 | 0.1940406 | 1.73929683 | 1.00E+00 |
| **F/n/-H/n/** | **1.14E+00** | **3.128315** | **0.195324** | **5.838987032** | **1.89E-07***** |
| **F/n/-L/n/** | **1.23E+00** | **3.4249587** | **0.1961279** | **6.276971202** | **1.24E-08***** |
| F/n/-R/n/ | 3.97E-01 | 1.4870457 | 0.1949862 | 2.034971778 | 1.00E+00 |
| H/n/-L/n/ | 9.06E-02 | 1.0948254 | 0.1904447 | 0.475701903 | 1.00E+00 |
| **H/n/-R/n/** | **-7.44E-01** | **0.4753504** | **0.1902379** | **-3.909332105** | **3.33E-03**** |
| **L/n/-R/n/** | **-8.34E-01** | **0.4341792** | **0.1910318** | **-4.367326252** | **4.53E-04***** |

**Table S3.11**

*Coefficients of the GLMM for Arousal of Vowels by Consonants in Nonce Words*

| Predictor | B (LogOdds) | Odds | SE B  (std. Error) | *Z* | *p* |
| --- | --- | --- | --- | --- | --- |
| Intercept (Vowel/i/:Consonant/t/) | 0.33260711 | 1.3945993 | 0.1033157 | 3.2193263 | 0.001284922** |
| /u/ vs. /i/ | 0.04466537 | 1.0456779 | 0.1336124 | 0.3342906 | 0.738160276 |
| /n/ vs. /t/ | -0.40259285 | 0.6685843 | 0.1327493 | -3.0327296 | 0.002423526** |
| /nu/ vs. /ti/ | 0.26938906 | 1.3091644 | 0.1880496 | 1.4325423 | 0.151988692 |

**Table S3.12**

*Pairwise Multiple Comparisons of the GLMM for Arousal among Vowels and Consonants in Nonce Words*

| Predictor | B (LogOdds) | Odds | SE B  (std. Error) | *Z* | *p* |
| --- | --- | --- | --- | --- | --- |
| /ti/-/tu/ | -0.04466537 | 0.9563174 | 0.1336124 | -0.3342906 | 1 |
| **/ti/-/ni/** | **0.40259285** | **1.4956978** | **0.1327493** | **3.0327296** | **0.024235262*** |
| /ti/-/nu/ | 0.08853841 | 1.0925762 | 0.13303 | 0.665552 | 1 |
| **/tu/-/ni/** | **0.44725822** | **1.5640181** | **0.133003** | **3.3627686** | **0.007716502**** |
| /tu/-/nu/ | 0.13320378 | 1.1424828 | 0.133263 | 0.9995557 | 1 |
| /ni/-/nu/ | -0.31405443 | 0.7304793 | 0.1323472 | -2.3729595 | 0.176461983 |

**Table S3.13**

*Coefficients of the GLMM for Arousal of Tones, Vowels by Consonants in Nonce Words*

| Predictor | B (LogOdds) | Odds | SE B  (std. Error) | *Z* | *p* |
| --- | --- | --- | --- | --- | --- |
| Intercept (ToneF:Vowel/i/:Consonant/t/) | 0.45391749 | 1.5744681 | 0.1865211 | 2.4335991 | 0.014949545* |
| H vs. F | 0.07015336 | 1.0726727 | 0.2648966 | 0.264833 | 0.791138141 |
| L vs. F | -0.73678028 | 0.4786526 | 0.2617511 | -2.8148127 | 0.004880567** |
| R vs. F | 0.14159122 | 1.1521056 | 0.2662075 | 0.5318829 | 0.594807087 |
| /u/ vs. /i/ | 0.61190497 | 1.8439407 | 0.2795697 | 2.1887386 | 0.028615842* |
| /n/ vs. /t/ | 0.14159122 | 1.1521056 | 0.2662075 | 0.5318829 | 0.594807087 |
| H:/u/ vs. F:/i/ | -1.25180764 | 0.2859874 | 0.383025 | -3.2682136 | 0.001082287** |
| L:/u/ vs. F:/i/ | -0.51136375 | 0.5996772 | 0.3810723 | -1.3419076 | 0.179625969 |
| R:/u/ vs. F:/i/ | -0.42615924 | 0.6530123 | 0.3906398 | -1.0909262 | 0.27530536 |
| H:/n/ vs. F:/t/ | -1.05050789 | 0.3497601 | 0.3748914 | -2.8021655 | 0.005076082** |
| L:/n/ vs. F:/t/ | -0.45423714 | 0.6349321 | 0.375055 | -1.2111213 | 0.225848907 |
| R:/n/ vs. F:/t/ | -0.62126811 | 0.5372627 | 0.3743149 | -1.6597474 | 0.096965275^.^ |
| /nu/ vs. /ti/ | -0.30841445 | 0.7346108 | 0.392982 | -0.7848056 | 0.432567541 |
| H:/nu/ vs. F:/ti/ | 1.05030015 | 2.858509 | 0.5394482 | 1.9469898 | 0.051535958^.^ |
| L:/nu/ vs. F:/ti/ | 0.55416614 | 1.7404891 | 0.5395756 | 1.0270408 | 0.304401245 |
| R:/nu/ vs. F:/ti/ | 0.63861513 | 1.8938563 | 0.5463622 | 1.1688493 | 0.242464347 |

**Table S3.14**

*Pairwise Multiple Comparisons of the GLMM for Arousal among Tones, Vowels, and Consonants in Nonce Words*

| Predictor | B (LogOdds) | Odds | SE B  (std. Error) | *Z* | *p* |
| --- | --- | --- | --- | --- | --- |
| F/ti/-H/ti/ | -7.02E-02 | 0.9322508 | 0.2648966 | -2.65E-01 | 1.00E+00 |
| F/ti/-L/ti/ | 7.37E-01 | 2.089198 | 0.2617511 | 2.81E+00 | 6.64E-01 |
| F/ti/-R/ti/ | -1.42E-01 | 0.867976 | 0.2662075 | -5.32E-01 | 1.00E+00 |
| F/ti/-F/tu/ | -6.12E-01 | 0.5423168 | 0.2795697 | -2.19E+00 | 1.00E+00 |
| F/ti/-H/tu/ | 5.70E-01 | 1.7678238 | 0.2606894 | 2.19E+00 | 1.00E+00 |
| F/ti/-L/tu/ | 6.36E-01 | 1.8893617 | 0.2610047 | 2.44E+00 | 1.00E+00 |
| F/ti/-R/tu/ | -3.27E-01 | 0.7208408 | 0.2704701 | -1.21E+00 | 1.00E+00 |
| F/ti/-F/ni/ | -1.42E-01 | 0.867976 | 0.2662075 | -5.32E-01 | 1.00E+00 |
| F/ti/-H/ni/ | 8.39E-01 | 2.3135041 | 0.2628444 | 3.19E+00 | 1.93E-01 |
| **F/ti/-L/ni/** | **1.05E+00** | **2.8560119** | **0.2662075** | **3.94E+00** | **1.10E-02*** |
| F/ti/-R/ni/ | 3.38E-01 | 1.4022606 | 0.2606894 | 1.30E+00 | 1.00E+00 |
| F/ti/-F/nu/ | -4.45E-01 | 0.6407719 | 0.2738419 | -1.63E+00 | 1.00E+00 |
| F/ti/-H/nu/ | 7.37E-01 | 2.089198 | 0.2617511 | 2.81E+00 | 6.64E-01 |
| F/ti/-L/nu/ | 7.03E-01 | 2.0200723 | 0.2614649 | 2.69E+00 | 9.74E-01 |
| F/ti/-R/nu/ | -1.78E-01 | 0.837059 | 0.2669417 | -6.66E-01 | 1.00E+00 |
| H/ti/-L/ti/ | 8.07E-01 | 2.2410256 | 0.2628757 | 3.07E+00 | 2.91E-01 |
| H/ti/-R/ti/ | -7.14E-02 | 0.9310541 | 0.2673134 | -2.67E-01 | 1.00E+00 |
| H/ti/-F/tu/ | -5.42E-01 | 0.5817284 | 0.2806229 | -1.93E+00 | 1.00E+00 |
| H/ti/-H/tu/ | 6.40E-01 | 1.8962963 | 0.2618186 | 2.44E+00 | 1.00E+00 |
| H/ti/-L/tu/ | 7.06E-01 | 2.0266667 | 0.2621325 | 2.69E+00 | 9.58E-01 |
| H/ti/-R/tu/ | -2.57E-01 | 0.7732262 | 0.2715586 | -9.47E-01 | 1.00E+00 |
| H/ti/-F/ni/ | -7.14E-02 | 0.9310541 | 0.2673134 | -2.67E-01 | 1.00E+00 |
| H/ti/-H/ni/ | 9.09E-01 | 2.4816327 | 0.2639643 | 3.44E+00 | 7.81E-02^.^ |
| **H/ti/-L/ni/** | **1.12E+00** | **3.0635659** | **0.2673134** | **4.19E+00** | **3.82E-03**** |
| H/ti/-R/ni/ | 4.08E-01 | 1.5041667 | 0.2618186 | 1.56E+00 | 1.00E+00 |
| H/ti/-F/nu/ | -3.75E-01 | 0.6873385 | 0.2749171 | -1.36E+00 | 1.00E+00 |
| H/ti/-H/nu/ | 8.07E-01 | 2.2410256 | 0.2628757 | 3.07E+00 | 2.91E-01 |
| H/ti/-L/nu/ | 7.73E-01 | 2.1668763 | 0.2625908 | 2.94E+00 | 4.39E-01 |
| H/ti/-R/nu/ | -1.08E-01 | 0.8978903 | 0.2680445 | -4.02E-01 | 1.00E+00 |
| L/ti/-R/ti/ | -8.78E-01 | 0.4154589 | 0.2641966 | -3.32E+00 | 1.20E-01 |
| **L/ti/-F/tu/** | **-1.35E+00** | **0.2595813** | **0.2776556** | **-4.86E+00** | **1.62E-04***** |
| L/ti/-H/tu/ | -1.67E-01 | 0.8461734 | 0.2586356 | -6.46E-01 | 1.00E+00 |
| L/ti/-L/tu/ | -1.01E-01 | 0.9043478 | 0.2589534 | -3.88E-01 | 1.00E+00 |
| **L/ti/-R/tu/** | **-1.06E+00** | **0.3450323** | **0.2684911** | **-3.96E+00** | **1.01E-02*** |
| L/ti/-F/ni/ | -8.78E-01 | 0.4154589 | 0.2641966 | -3.32E+00 | 1.20E-01 |
| L/ti/-H/ni/ | 1.02E-01 | 1.1073647 | 0.2608075 | 3.91E-01 | 1.00E+00 |
| L/ti/-L/ni/ | 3.13E-01 | 1.3670374 | 0.2641966 | 1.18E+00 | 1.00E+00 |
| L/ti/-R/ni/ | -3.99E-01 | 0.6711957 | 0.2586356 | -1.54E+00 | 1.00E+00 |
| **L/ti/-F/nu/** | **-1.18E+00** | **0.3067071** | **0.2718875** | **-4.35E+00** | **1.88E-03**** |
| L/ti/-H/nu/ | -1.76E-14 | 1 | 0.2597057 | -6.79E-14 | 1.00E+00 |
| L/ti/-L/nu/ | -3.36E-02 | 0.9669128 | 0.2594173 | -1.30E-01 | 1.00E+00 |
| L/ti/-R/nu/ | -9.15E-01 | 0.4006604 | 0.2649363 | -3.45E+00 | 7.56E-02^.^ |
| R/ti/-F/tu/ | -4.70E-01 | 0.6248062 | 0.2818606 | -1.67E+00 | 1.00E+00 |
| R/ti/-H/tu/ | 7.11E-01 | 2.0367197 | 0.2631448 | 2.70E+00 | 9.34E-01 |
| R/ti/-L/tu/ | 7.78E-01 | 2.1767442 | 0.2634571 | 2.95E+00 | 4.29E-01 |
| R/ti/-R/tu/ | -1.86E-01 | 0.8304847 | 0.2728375 | -6.81E-01 | 1.00E+00 |
| R/ti/-F/ni/ | -5.97E-15 | 1 | 0.2686124 | -2.22E-14 | 1.00E+00 |
| **R/ti/-H/ni/** | **9.80E-01** | **2.665401** | **0.2652798** | **3.70E+00** | **2.98E-02*** |
| **R/ti/-L/ni/** | **1.19E+00** | **3.2904273** | **0.2686124** | **4.43E+00** | **1.26E-03**** |
| R/ti/-R/ni/ | 4.80E-01 | 1.6155523 | 0.2631448 | 1.82E+00 | 1.00E+00 |
| R/ti/-F/nu/ | -3.03E-01 | 0.7382369 | 0.2761804 | -1.10E+00 | 1.00E+00 |
| R/ti/-H/nu/ | 8.78E-01 | 2.4069767 | 0.2641966 | 3.32E+00 | 1.20E-01 |
| R/ti/-L/nu/ | 8.45E-01 | 2.3273366 | 0.2639131 | 3.20E+00 | 1.86E-01 |
| R/ti/-R/nu/ | -3.63E-02 | 0.9643803 | 0.2693401 | -1.35E-01 | 1.00E+00 |
| **F/tu/-H/tu/** | **1.18E+00** | **3.2597623** | **0.2766549** | **4.27E+00** | **2.64E-03**** |
| **F/tu/-L/tu/** | **1.25E+00** | **3.483871** | **0.276952** | **4.51E+00** | **8.95E-04***** |
| F/tu/-R/tu/ | 2.85E-01 | 1.3291877 | 0.2858899 | 9.95E-01 | 1.00E+00 |
| F/tu/-F/ni/ | 4.70E-01 | 1.6004963 | 0.2818606 | 1.67E+00 | 1.00E+00 |
| **F/tu/-H/ni/** | **1.45E+00** | **4.2659645** | **0.2786865** | **5.21E+00** | **2.63E-05***** |
| **F/tu/-L/ni/** | **1.66E+00** | **5.2663166** | **0.2818606** | **5.89E+00** | **5.12E-07***** |
| F/tu/-R/ni/ | 9.50E-01 | 2.5856855 | 0.2766549 | 3.43E+00 | 8.09E-02^.^ |
| F/tu/-F/nu/ | 1.67E-01 | 1.1815454 | 0.289082 | 5.77E-01 | 1.00E+00 |
| **F/tu/-H/nu/** | **1.35E+00** | **3.8523573** | **0.2776556** | **4.86E+00** | **1.62E-04***** |
| **F/tu/-L/nu/** | **1.32E+00** | **3.7248935** | **0.2773858** | **4.74E+00** | **2.89E-04***** |
| F/tu/-R/nu/ | 4.34E-01 | 1.5434871 | 0.2825541 | 1.54E+00 | 1.00E+00 |
| H/tu/-L/tu/ | 6.65E-02 | 1.06875 | 0.2578802 | 2.58E-01 | 1.00E+00 |
| H/tu/-R/tu/ | -8.97E-01 | 0.407756 | 0.2674562 | -3.35E+00 | 1.08E-01 |
| H/tu/-F/ni/ | -7.11E-01 | 0.4909856 | 0.2631448 | -2.70E+00 | 9.34E-01 |
| H/tu/-H/ni/ | 2.69E-01 | 1.3086735 | 0.259742 | 1.04E+00 | 1.00E+00 |
| H/tu/-L/ni/ | 4.80E-01 | 1.6155523 | 0.2631448 | 1.82E+00 | 1.00E+00 |
| H/tu/-R/ni/ | -2.32E-01 | 0.7932129 | 0.2575611 | -8.99E-01 | 1.00E+00 |
| **H/tu/-F/nu/** | **-1.01E+00** | **0.3624637** | **0.2708656** | **-3.75E+00** | **2.44E-02*** |
| H/tu/-H/nu/ | 1.67E-01 | 1.1817909 | 0.2586356 | 6.46E-01 | 1.00E+00 |
| H/tu/-L/nu/ | 1.33E-01 | 1.1426887 | 0.258346 | 5.16E-01 | 1.00E+00 |
| H/tu/-R/nu/ | -7.48E-01 | 0.4734968 | 0.2638875 | -2.83E+00 | 6.27E-01 |
| **L/tu/-R/tu/** | **-9.64E-01** | **0.3815261** | **0.2677635** | **-3.60E+00** | **4.35E-02*** |
| L/tu/-F/ni/ | -7.78E-01 | 0.4594017 | 0.2634571 | -2.95E+00 | 4.29E-01 |
| L/tu/-H/ni/ | 2.03E-01 | 1.2244898 | 0.2600584 | 7.79E-01 | 1.00E+00 |
| L/tu/-L/ni/ | 4.13E-01 | 1.5116279 | 0.2634571 | 1.57E+00 | 1.00E+00 |
| L/tu/-R/ni/ | -2.98E-01 | 0.7421875 | 0.2578802 | -1.16E+00 | 1.00E+00 |
| **L/tu/-F/nu/** | **-1.08E+00** | **0.3391473** | **0.271169** | **-3.99E+00** | **9.08E-03**** |
| L/tu/-H/nu/ | 1.01E-01 | 1.1057692 | 0.2589534 | 3.88E-01 | 1.00E+00 |
| L/tu/-L/nu/ | 6.69E-02 | 1.0691824 | 0.2586641 | 2.59E-01 | 1.00E+00 |
| L/tu/-R/nu/ | -8.14E-01 | 0.443038 | 0.2641989 | -3.08E+00 | 2.80E-01 |
| R/tu/-F/ni/ | 1.86E-01 | 1.2041161 | 0.2728375 | 6.81E-01 | 1.00E+00 |
| **R/tu/-H/ni/** | **1.17E+00** | **3.2094522** | **0.2695571** | **4.33E+00** | **2.07E-03**** |
| **R/tu/-L/ni/** | **1.38E+00** | **3.9620563** | **0.2728375** | **5.05E+00** | **6.13E-05***** |
| R/tu/-R/ni/ | 6.65E-01 | 1.9453125 | 0.2674562 | 2.49E+00 | 1.00E+00 |
| R/tu/-F/nu/ | -1.18E-01 | 0.8889229 | 0.2802914 | -4.20E-01 | 1.00E+00 |
| **R/tu/-H/nu/** | **1.06E+00** | **2.8982794** | **0.2684911** | **3.96E+00** | **1.01E-02*** |
| **R/tu/-L/nu/** | **1.03E+00** | **2.8023833** | **0.2682122** | **3.84E+00** | **1.66E-02*** |
| R/tu/-R/nu/ | 1.49E-01 | 1.1612258 | 0.2735538 | 5.46E-01 | 1.00E+00 |
| **F/ni/-H/ni/** | **9.80E-01** | **2.665401** | **0.2652798** | **3.70E+00** | **2.98E-02*** |
| **F/ni/-L/ni/** | **1.19E+00** | **3.2904273** | **0.2686124** | **4.43E+00** | **1.26E-03**** |
| F/ni/-R/ni/ | 4.80E-01 | 1.6155523 | 0.2631448 | 1.82E+00 | 1.00E+00 |
| F/ni/-F/nu/ | -3.03E-01 | 0.7382369 | 0.2761804 | -1.10E+00 | 1.00E+00 |
| F/ni/-H/nu/ | 8.78E-01 | 2.4069767 | 0.2641966 | 3.32E+00 | 1.20E-01 |
| F/ni/-L/nu/ | 8.45E-01 | 2.3273366 | 0.2639131 | 3.20E+00 | 1.86E-01 |
| F/ni/-R/nu/ | -3.63E-02 | 0.9643803 | 0.2693401 | -1.35E-01 | 1.00E+00 |
| H/ni/-L/ni/ | 2.11E-01 | 1.2344961 | 0.2652798 | 7.94E-01 | 1.00E+00 |
| H/ni/-R/ni/ | -5.01E-01 | 0.6061198 | 0.259742 | -1.93E+00 | 1.00E+00 |
| **H/ni/-F/nu/** | **-1.28E+00** | **0.2769703** | **0.2729402** | **-4.70E+00** | **3.47E-04***** |
| H/ni/-H/nu/ | -1.02E-01 | 0.9030449 | 0.2608075 | -3.91E-01 | 1.00E+00 |
| H/ni/-L/nu/ | -1.36E-01 | 0.8731656 | 0.2605204 | -5.21E-01 | 1.00E+00 |
| **H/ni/-R/nu/** | **-1.02E+00** | **0.3618143** | **0.2660165** | **-3.82E+00** | **1.80E-02*** |
| L/ni/-R/ni/ | -7.11E-01 | 0.4909856 | 0.2631448 | -2.70E+00 | 9.34E-01 |
| **L/ni/-F/nu/** | **-1.49E+00** | **0.224359** | **0.2761804** | **-5.41E+00** | **8.51E-06***** |
| L/ni/-H/nu/ | -3.13E-01 | 0.7315089 | 0.2641966 | -1.18E+00 | 1.00E+00 |
| L/ni/-L/nu/ | -3.46E-01 | 0.7073053 | 0.2639131 | -1.31E+00 | 1.00E+00 |
| **L/ni/-R/nu/** | **-1.23E+00** | **0.2930867** | **0.2693401** | **-4.56E+00** | **7.07E-04***** |
| R/ni/-F/nu/ | -7.83E-01 | 0.4569563 | 0.2708656 | -2.89E+00 | 5.22E-01 |
| R/ni/-H/nu/ | 3.99E-01 | 1.4898785 | 0.2586356 | 1.54E+00 | 1.00E+00 |
| R/ni/-L/nu/ | 3.65E-01 | 1.4405826 | 0.258346 | 1.41E+00 | 1.00E+00 |
| R/ni/-R/nu/ | -5.16E-01 | 0.5969354 | 0.2638875 | -1.96E+00 | 1.00E+00 |
| **F/nu/-H/nu/** | **1.18E+00** | **3.2604396** | **0.2718875** | **4.35E+00** | **1.88E-03**** |
| **F/nu/-L/nu/** | **1.15E+00** | **3.1525606** | **0.271612** | **4.23E+00** | **3.22E-03**** |
| F/nu/-R/nu/ | 2.67E-01 | 1.3063291 | 0.2768881 | 9.65E-01 | 1.00E+00 |
| H/nu/-L/nu/ | -3.36E-02 | 0.9669128 | 0.2594173 | -1.30E-01 | 1.00E+00 |
| H/nu/-R/nu/ | -9.15E-01 | 0.4006604 | 0.2649363 | -3.45E+00 | 7.56E-02^.^ |
| L/nu/-R/nu/ | -8.81E-01 | 0.4143708 | 0.2646536 | -3.33E+00 | 1.19E-01 |

**Table S3.15**

*Post-hoc Power of the Arousal Models in Nonce words*

| Model | Predictor | Effect size/Odds | *Power* |
| --- | --- | --- | --- |
| Arousal ~ Tone | Intercept (Tone F) | 2.1927691 | 1 |
|  | H vs. F | 0.4249541 | 1 |
|  | L vs. F | 0.323026 | 1 |
|  | R vs. F | 0.7937348 | 1 |
| Arousal ~ Vowel | Intercept (Vowel /i/) | 1.138895 | 1 |
|  | /u/ vs. /i/ | 1.197283 | 1 |
| Arousal ~ Tone * Vowel | Intercept (ToneF:Vowel/i/) | 1.7416325 | 1 |
|  | H vs. F | 0.6155647 | 1 |
|  | L vs. F | 0.3612886 | 1 |
|  | R vs. F | 0.8323272 | 1 |
|  | /u/ vs. /i/ | 1.6180304 | 1 |
|  | H:/u/ vs. F:/i/ | 0.4665391 | 1 |
|  | L:/u/ vs. F:/i/ | 0.7809175 | 1 |
|  | R:/u/ vs. F:/i/ | 0.8993972 | 1 |
| Arousal ~ Consonant | Intercept (Consonant /t/) | 1.4257893 | 1 |
|  | /n/ vs. /t/ | 0.7647636 | 1 |
| Arousal ~ Tone * Consonant | Intercept (ToneF:Consonant/t/) | 2.1949277 | 1 |
|  | H vs. F | 0.5623177 | 1 |
|  | L vs. F | 0.3558824 | 1 |
|  | R vs. F | 0.9424345 | 1 |
|  | /n/ vs. /t/ | 1.0000083 | 1 |
|  | H:/n/ vs. F:/t/ | 0.5684703 | 1 |
|  | L:/n/ vs. F:/t/ | 0.8204235 | 1 |
|  | R:/n/ vs. F:/t/ | 0.7135502 | 1 |
| Arousal ~ Vowel * Consonant | Intercept (Vowel/i/:Consonant/t/) | 1.3945993 | 1 |
|  | /u/ vs. /i/ | 1.0456779 | 1 |
|  | /n/ vs. /t/ | 0.6685843 | 1 |
|  | /nu/ vs. /ti/ | 1.3091644 | 1 |
| Arousal ~ Tone * Vowel * Consonant | Intercept (ToneF:Vowel/i/:Consonant/t/) | 1.5744681 | 1 |
|  | H vs. F | 1.0726727 | 1 |
|  | L vs. F | 0.4786526 | 1 |
|  | R vs. F | 1.1521056 | 1 |
|  | /u/ vs. /i/ | 1.8439407 | 1 |
|  | /n/ vs. /t/ | 1.1521056 | 1 |
|  | H:/u/ vs. F:/i/ | 0.2859874 | 1 |
|  | L:/u/ vs. F:/i/ | 0.5996772 | 1 |
|  | R:/u/ vs. F:/i/ | 0.6530123 | 1 |
|  | H:/n/ vs. F:/t/ | 0.3497601 | 1 |
|  | L:/n/ vs. F:/t/ | 0.6349321 | 1 |
|  | R:/n/ vs. F:/t/ | 0.5372627 | 1 |
|  | /nu/ vs. /ti/ | 0.7346108 | 1 |
|  | H:/nu/ vs. F:/ti/ | 2.858509 | 1 |
|  | L:/nu/ vs. F:/ti/ | 1.7404891 | 1 |
|  | R:/nu/ vs. F:/ti/ | 1.8938563 | 1 |

# **4 Valence in CVL Nonce Word Dataset**

**Table S4.1**

*Coefficients of the GLMM for Valence of Tones in Nonce Words*

| Predictor | B (LogOdds) | Odds | SE B  (std. Error) | *Z* | *p* |
| --- | --- | --- | --- | --- | --- |
| Intercept (Tone F) | 8.72E-01 | 2.3913175 | 0.1022446 | 8.53E+00 | 1.50E-17*** |
| H vs. F | -1.38E+00 | 0.2509094 | 0.1324704 | -1.04E+01 | 1.67E-25*** |
| L vs. F | 3.57E-06 | 1.0000036 | 0.1348062 | 2.65E-05 | 1.00E+00 |
| R vs. F | -1.41E+00 | 0.2448151 | 0.132683 | -1.06E+01 | 2.79E-26*** |

**Table S4.2**

*Pairwise Multiple Comparisons of the GLMM for Valence among Tones in Nonce Words*

| Predictor | B (LogOdds) | Odds | SE B  (std. Error) | *Z* | *p* |
| --- | --- | --- | --- | --- | --- |
| **F-H** | **1.38E+00** | **3.985503** | **0.1324704** | **1.04E+01** | **1.67E-24***** |
| F-L | -3.57E-06 | 0.9999964 | 0.1348062 | -2.65E-05 | 1.00E+00 |
| **F-R** | **1.41E+00** | **4.0847157** | **0.132683** | **1.06E+01** | **2.79E-25***** |
| **H-L** | **-1.38E+00** | **0.2509085** | **0.1324746** | **-1.04E+01** | **1.68E-24***** |
| H-R | 2.46E-02 | 1.0248934 | 0.1280501 | 1.92E-01 | 1.00E+00 |
| **L-R** | **1.41E+00** | **4.0847303** | **0.1326872** | **1.06E+01** | **2.80E-25***** |

**Table S4.3**

*Coefficients of the GLMM for Valence of Vowels in Nonce Words*

| Predictor | B (LogOdds) | Odds | SE B  (std. Error) | *Z* | *p* |
| --- | --- | --- | --- | --- | --- |
| Intercept (Vowel /i/) | 0.000164503 | 1.000165 | 0.06765683 | 0.002431426 | 0.998060005 |
| /u/ vs. /i/ | 0.310131575 | 1.363605 | 0.08772836 | 3.53513469 | 0.000407567*** |

**Table S4.4**

*Pairwise Multiple Comparisons of the GLMM for Valence between Vowels in Nonce Words*

| Predictor | B (LogOdds) | Odds | SE B  (std. Error) | *Z* | *p* |
| --- | --- | --- | --- | --- | --- |
| **/i/-/u/** | **-0.3101316** | **0.7333505** | **0.08772836** | **-3.535135** | **0.001222702**** |

**Table S4.5**

*Coefficients of the GLMM for Valence of Tones by Vowels in Nonce Words*

| Predictor | B (LogOdds) | Odds | SE B  (std. Error) | *Z* | *p* |
| --- | --- | --- | --- | --- | --- |
| Intercept (ToneF:Vowel/i/) | 0.62718921 | 1.8723404 | 0.1277502 | 4.9094971 | 9.13E-07*** |
| H vs. F | -1.27073945 | 0.280624 | 0.1808937 | -7.0247873 | 2.14E-12*** |
| L vs. F | 0.09948066 | 1.1045971 | 0.182149 | 0.54615 | 5.85E-01 |
| R vs. F | -1.33704987 | 0.2626193 | 0.1818816 | -7.3512095 | 1.96E-13*** |
| /u/ vs. /i/ | 0.44200919 | 1.55583 | 0.1891771 | 2.3364842 | 1.95E-02* |
| H:/u/ vs. F:/i/ | -0.14255511 | 0.8671398 | 0.2597076 | -0.5489062 | 5.83E-01 |
| L:/u/ vs. F:/i/ | -0.21316762 | 0.8080207 | 0.2666601 | -0.7993983 | 4.24E-01 |
| R:/u/ vs. F:/i/ | -0.06100652 | 0.9408171 | 0.2603224 | -0.2343499 | 8.15E-01 |

**Table S4.6**

*Pairwise Multiple Comparisons of the GLMM for Valence among Tones and Vowels in Nonce Words*

| Predictor | B (LogOdds) | Odds | SE B  (std. Error) | *Z* | *p* |
| --- | --- | --- | --- | --- | --- |
| **F/i/-H/i/** | **1.27073945** | **3.5634866** | **0.1808937** | **7.02478731** | **7.72E-11***** |
| F/i/-L/i/ | -0.09948066 | 0.9053075 | 0.182149 | -0.54615001 | 1.00E+00 |
| **F/i/-R/i/** | **1.33704987** | **3.8077934** | **0.1818816** | **7.35120952** | **7.07E-12***** |
| F/i/-F/u/ | -0.44200919 | 0.6427437 | 0.1891771 | -2.33648418 | 7.01E-01 |
| **F/i/-H/u/** | **0.97128537** | **2.6413374** | **0.1777014** | **5.46582759** | **1.66E-06***** |
| F/i/-L/u/ | -0.32832223 | 0.7201309 | 0.1864983 | -1.76045675 | 1.00E+00 |
| **F/i/-R/u/** | **0.9560472** | **2.6013933** | **0.1775925** | **5.38337591** | **2.63E-06***** |
| **H/i/-L/i/** | **-1.37022011** | **0.254051** | **0.1823747** | **-7.5132133** | **2.08E-12***** |
| H/i/-R/i/ | 0.06631042 | 1.0685584 | 0.1821077 | 0.36412752 | 1.00E+00 |
| **H/i/-F/u/** | **-1.71274864** | **0.1803693** | **0.1893944** | **-9.04328989** | **5.47E-18***** |
| H/i/-H/u/ | -0.29945408 | 0.7412228 | 0.1779328 | -1.68296149 | 1.00E+00 |
| **H/i/-L/u/** | **-1.59906168** | **0.202086** | **0.1867188** | **-8.56400967** | **3.92E-16***** |
| H/i/-R/u/ | -0.31469225 | 0.7300135 | 0.177824 | -1.7696835 | 1.00E+00 |
| **L/i/-R/i/** | **1.43653053** | **4.2060776** | **0.1833547** | **7.83470825** | **1.69E-13***** |
| L/i/-F/u/ | -0.34252853 | 0.7099729 | 0.1905938 | -1.79716558 | 1.00E+00 |
| **L/i/-H/u/** | **1.07076603** | **2.9176136** | **0.1792089** | **5.97496113** | **8.29E-08***** |
| L/i/-L/u/ | -0.22884157 | 0.7954545 | 0.1879352 | -1.217662 | 1.00E+00 |
| **L/i/-R/u/** | **1.05552786** | **2.8734916** | **0.1791009** | **5.89348291** | **1.36E-07***** |
| **R/i/-F/u/** | **-1.77905906** | **0.1687969** | **0.1903383** | **-9.34682829** | **3.25E-19***** |
| R/i/-H/u/ | -0.3657645 | 0.6936661 | 0.1789371 | -2.04409508 | 1.00E+00 |
| **R/i/-L/u/** | **-1.66537211** | **0.1891203** | **0.1876761** | **-8.87365002** | **2.55E-17***** |
| R/i/-R/u/ | -0.38100267 | 0.6831761 | 0.178829 | -2.13054245 | 1.00E+00 |
| **F/u/-H/u/** | **1.41329457** | **4.109472** | **0.1863479** | **7.5841733** | **1.20E-12***** |
| F/u/-L/u/ | 0.11368696 | 1.1204013 | 0.1947546 | 0.58374465 | 1.00E+00 |
| **F/u/-R/u/** | **1.39805639** | **4.0473259** | **0.186244** | **7.50658472** | **2.18E-12***** |
| **H/u/-L/u/** | **-1.29960761** | **0.2726388** | **0.1836279** | **-7.07739821** | **5.29E-11***** |
| H/u/-R/u/ | -0.01523818 | 0.9848773 | 0.1745757 | -0.08728693 | 1.00E+00 |
| **L/u/-R/u/** | **1.28436943** | **3.6123894** | **0.1835225** | **6.99843157** | **9.32E-11***** |

**Table S4.7**

*Coefficients of the GLMM for Valence of Consonants in Nonce Words*

| Predictor | B (LogOdds) | Odds | SE B  (std. Error) | *Z* | *p* |
| --- | --- | --- | --- | --- | --- |
| Intercept (Consonant /t/) | 0.09518464 | 1.099862 | 0.06756786 | 1.408727 | 0.158916 |
| /n/ vs. /t/ | 0.1184281 | 1.125726 | 0.08743713 | 1.354437 | 0.1755969 |

**Table S4.8**

*Pairwise Multiple Comparisons of the GLMM for Valence between Consonants in Nonce Words*

| Predictor | B (LogOdds) | Odds | SE B  (std. Error) | *Z* | *p* |
| --- | --- | --- | --- | --- | --- |
| /t/-/n/ | -0.1184281 | 0.8883157 | 0.08743713 | -1.354437 | 0.5267908 |

**Table S4.9**

*Coefficients of the GLMM for Valence of Tones by Consonants in Nonce Words*

| Predictor | B (LogOdds) | Odds | SE B  (std. Error) | *Z* | *p* |
| --- | --- | --- | --- | --- | --- |
| Intercept (ToneF:Consonant/t/) | 0.70458793 | 2.0230129 | 0.1361955 | 5.1733569 | 2.30E-07*** |
| H vs. F | -1.40845978 | 0.2445196 | 0.1863304 | -7.5589376 | 4.06E-14*** |
| L vs. F | 0.30805917 | 1.3607815 | 0.1908641 | 1.6140237 | 1.07E-01 |
| R vs. F | -1.27340604 | 0.2798767 | 0.1844908 | -6.9022724 | 5.12E-12*** |
| /n/ vs. /t/ | 0.34685907 | 1.4146174 | 0.1917264 | 1.8091359 | 7.04E-02^.^ |
| H:/n/ vs. F:/t/ | 0.03060611 | 1.0310793 | 0.2641636 | 0.1158604 | 9.08E-01 |
| L:/n/ vs. F:/t/ | -0.62033731 | 0.537763 | 0.2709094 | -2.2898332 | 2.20E-02* |
| R:/n/ vs. F:/t/ | -0.28101643 | 0.7550159 | 0.2639727 | -1.0645662 | 2.87E-01 |

**Table S4.10**

*Pairwise Multiple Comparisons of the GLMM for Valence among Tones and Consonants in Nonce Word*

| Predictor | B (LogOdds) | Odds | SE B  (std. Error) | *Z* | *p* |
| --- | --- | --- | --- | --- | --- |
| **F/t/-H/t/** | **1.40845978** | **4.0896516** | **0.1863304** | **7.5589376** | **1.46E-12***** |
| F/t/-L/t/ | -0.30805917 | 0.7348718 | 0.1908641 | -1.6140237 | 1.00E+00 |
| **F/t/-R/t/** | **1.27340604** | **3.5730017** | **0.1844908** | **6.9022724** | **1.84E-10***** |
| F/t/-F/n/ | -0.34685907 | 0.7069049 | 0.1917264 | -1.8091359 | 1.00E+00 |
| **F/t/-H/n/** | **1.03099459** | **2.8038531** | **0.1821354** | **5.6605941** | **5.43E-07***** |
| F/t/-L/n/ | -0.03458094 | 0.9660101 | 0.1859193 | -0.1859998 | 1.00E+00 |
| **F/t/-R/n/** | **1.2075634** | **3.3453235** | **0.1837206** | **6.5728262** | **1.78E-09***** |
| **H/t/-L/t/** | **-1.71651895** | **0.1796906** | **0.1920297** | **-8.9388199** | **1.42E-17***** |
| H/t/-R/t/ | -0.13505374 | 0.873669 | 0.1838622 | -0.7345378 | 1.00E+00 |
| **H/t/-F/n/** | **-1.75531885** | **0.1728521** | **0.1929132** | **-9.0990062** | **3.28E-18***** |
| H/t/-H/n/ | -0.37746519 | 0.6855971 | 0.1818063 | -2.0761945 | 1.00E+00 |
| **H/t/-L/n/** | **-1.44304072** | **0.2362084** | **0.1868494** | **-7.7230169** | **4.09E-13***** |
| H/t/-R/n/ | -0.20089638 | 0.8179972 | 0.1831802 | -1.0967144 | 1.00E+00 |
| **L/t/-R/t/** | **1.58146522** | **4.8620746** | **0.1902121** | **8.3142208** | **3.32E-15***** |
| L/t/-F/n/ | -0.0387999 | 0.9619432 | 0.1969005 | -0.1970533 | 1.00E+00 |
| **L/t/-H/n/** | **1.33905376** | **3.8154315** | **0.1878872** | **7.1269007** | **3.70E-11***** |
| L/t/-L/n/ | 0.27347823 | 1.3145287 | 0.1913273 | 1.4293737 | 1.00E+00 |
| **L/t/-R/n/** | **1.51562258** | **4.5522544** | **0.1894641** | **7.9995236** | **4.50E-14***** |
| **R/t/-F/n/** | **-1.62026512** | **0.1978462** | **0.1911002** | **-8.4786174** | **8.20E-16***** |
| R/t/-H/n/ | -0.24241145 | 0.7847332 | 0.1800329 | -1.3464845 | 1.00E+00 |
| **R/t/-L/n/** | **-1.30798698** | **0.2703638** | **0.1850085** | **-7.0698759** | **5.58E-11***** |
| R/t/-R/n/ | -0.06584264 | 0.9362782 | 0.1814429 | -0.3628836 | 1.00E+00 |
| **F/n/-H/n/** | **1.37785366** | **3.9663793** | **0.1887848** | **7.2985407** | **1.05E-11***** |
| F/n/-L/n/ | 0.31227813 | 1.3665347 | 0.1921808 | 1.6249188 | 1.00E+00 |
| **F/n/-R/n/** | **1.55442248** | **4.7323527** | **0.1903595** | **8.1657219** | **1.15E-14***** |
| **H/n/-L/n/** | **-1.06557553** | **0.3445295** | **0.1826634** | **-5.8335465** | **1.95E-07***** |
| H/n/-R/n/ | 0.17656881 | 1.1931165 | 0.1793122 | 0.9847004 | 1.00E+00 |
| **L/n/-R/n/** | **1.24214434** | **3.4630314** | **0.1842486** | **6.7416763** | **5.64E-10***** |

**Table S4.11**

*Coefficients of the GLMM for Valence of Vowel by Consonants in Nonce Word*

| Predictor | B (LogOdds) | Odds | SE B  (std. Error) | *Z* | *p* |
| --- | --- | --- | --- | --- | --- |
| Intercept (Vowel/i/:Consonant/t/) | -0.04545305 | 0.9555645 | 0.09157533 | -0.4963461 | 0.61965027 |
| /u/ vs. /i/ | 0.28230441 | 1.3261824 | 0.12375011 | 2.2812458 | 0.02253391* |
| /n/ vs. /t/ | 0.09124422 | 1.0955365 | 0.1233359 | 0.7398026 | 0.45941977 |
| /nu/ vs. /ti/ | 0.05649121 | 1.0581173 | 0.17542298 | 0.3220285 | 0.74743107 |

**Table S4.12**

*Pairwise Multiple Comparisons of the GLMM for Valence among Vowels and Consonants in Nonce Word*

| Predictor | B (LogOdds) | Odds | SE B  (std. Error) | *Z* | *p* |
| --- | --- | --- | --- | --- | --- |
| /ti/-/tu/ | -0.28230441 | 0.7540441 | 0.1237501 | -2.2812458 | 0.225339083 |
| /ti/-/ni/ | -0.09124422 | 0.9127948 | 0.1233359 | -0.7398026 | 1 |
| **/ti/-/nu/** | **-0.43003984** | **0.6504832** | **0.1244549** | **-3.455387** | **0.005495035**** |
| /tu/-/ni/ | 0.19106019 | 1.2105323 | 0.1237287 | 1.544186 | 1 |
| /tu/-/nu/ | -0.14773543 | 0.8626593 | 0.1247581 | -1.1841755 | 1 |
| /ni/-/nu/ | -0.33879562 | 0.7126281 | 0.1244212 | -2.722973 | 0.064697344^.^ |

**Table S4.13**

*Coefficients of the GLMM for Valence of Tone, Vowel by Consonants in Nonce Word*

| Predictor | B (LogOdds) | Odds | SE B  (std. Error) | *Z* | *p* |
| --- | --- | --- | --- | --- | --- |
| Intercept (ToneF:Vowel/i/:Consonant/t/) | 0.43642733 | 1.5471698 | 0.1762471 | 2.47622397 | 1.33E-02* |
| H vs. F | -1.4104769 | 0.2440269 | 0.2613314 | -5.39727305 | 6.77E-08*** |
| L vs. F | 0.39329538 | 1.481856 | 0.2570828 | 1.52983945 | 1.26E-01 |
| R vs. F | -0.9354185 | 0.3924216 | 0.2501513 | -3.73941161 | 1.84E-04*** |
| /u/ vs. /i/ | 0.50069749 | 1.6498716 | 0.2601693 | 1.92450642 | 5.43E-02^.^ |
| /n/ vs. /t/ | 0.39329538 | 1.481856 | 0.2570828 | 1.52983945 | 1.26E-01 |
| H:/u/ vs. F:/i/ | 0.06788697 | 1.0702443 | 0.3684879 | 0.18423123 | 8.54E-01 |
| L:/u/ vs. F:/i/ | -0.20195495 | 0.8171317 | 0.3779199 | -0.53438558 | 5.93E-01 |
| R:/u/ vs. F:/i/ | -0.59641343 | 0.5507835 | 0.3626682 | -1.64451521 | 1.00E-01 |
| H:/n/ vs. F:/t/ | 0.23665802 | 1.2670078 | 0.3658387 | 0.64689165 | 5.18E-01 |
| L:/n/ vs. F:/t/ | -0.59582888 | 0.5511056 | 0.3657325 | -1.62913828 | 1.03E-01 |
| R:/n/ vs. F:/t/ | -0.83142903 | 0.4354266 | 0.366373 | -2.26935125 | 2.32E-02* |
| /nu/ vs. /ti/ | -0.12001651 | 0.8869058 | 0.3802499 | -0.31562537 | 7.52E-01 |
| H:/nu/ vs. F:/ti/ | -0.3878342 | 0.6785248 | 0.5229044 | -0.74169242 | 4.58E-01 |
| L:/nu/ vs. F:/ti/ | -0.01098537 | 0.9890748 | 0.5356564 | -0.02050824 | 9.84E-01 |
| R:/nu/ vs. F:/ti/ | 1.07874929 | 2.9409989 | 0.5241426 | 2.05812166 | 3.96E-02* |

**Table S4.14**

*Pairwise Multiple Comparisons of the GLMM for Valence among Tones, Vowels, and Consonants in Nonce Word*

| Predictor | B (LogOdds) | Odds | SE B  (std. Error) | *Z* | *p* |
| --- | --- | --- | --- | --- | --- |
| **F/ti/-H/ti/** | **1.41E+00** | **4.0979092** | **0.2613314** | **5.40E+00** | **9.20E-06***** |
| F/ti/-L/ti/ | -3.93E-01 | 0.6748294 | 0.2570828 | -1.53E+00 | 1.00E+00 |
| **F/ti/-R/ti/** | **9.35E-01** | **2.5482797** | **0.2501513** | **3.74E+00** | **2.51E-02*** |
| F/ti/-F/tu/ | -5.01E-01 | 0.6061078 | 0.2601693 | -1.92E+00 | 1.00E+00 |
| F/ti/-H/tu/ | 8.42E-01 | 2.3207547 | 0.2488518 | 3.38E+00 | 9.75E-02^.^ |
| F/ti/-L/tu/ | -6.92E-01 | 0.5005549 | 0.2667769 | -2.59E+00 | 1.00E+00 |
| **F/ti/-R/tu/** | **1.03E+00** | **2.8042453** | **0.251775** | **4.10E+00** | **5.73E-03**** |
| F/ti/-F/ni/ | -3.93E-01 | 0.6748294 | 0.2570828 | -1.53E+00 | 1.00E+00 |
| F/ti/-H/ni/ | 7.81E-01 | 2.1826146 | 0.24815 | 3.15E+00 | 2.26E-01 |
| F/ti/-L/ni/ | -1.91E-01 | 0.8263293 | 0.2523951 | -7.56E-01 | 1.00E+00 |
| **F/ti/-R/ni/** | **1.37E+00** | **3.9493545** | **0.2601693** | **5.28E+00** | **1.76E-05***** |
| F/ti/-F/nu/ | -7.74E-01 | 0.4611756 | 0.2700675 | -2.87E+00 | 5.66E-01 |
| F/ti/-H/nu/ | 7.20E-01 | 2.0540013 | 0.2475711 | 2.91E+00 | 4.96E-01 |
| F/ti/-L/nu/ | -3.59E-01 | 0.6987219 | 0.2561742 | -1.40E+00 | 1.00E+00 |
| F/ti/-R/nu/ | 5.11E-01 | 1.6661829 | 0.2464414 | 2.07E+00 | 1.00E+00 |
| **H/ti/-L/ti/** | **-1.80E+00** | **0.1646765** | **0.2688114** | **-6.71E+00** | **2.64E-09***** |
| H/ti/-R/ti/ | -4.75E-01 | 0.6218487 | 0.2621901 | -1.81E+00 | 1.00E+00 |
| **H/ti/-F/tu/** | **-1.91E+00** | **0.1479066** | **0.2717647** | **-7.03E+00** | **2.76E-10***** |
| H/ti/-H/tu/ | -5.69E-01 | 0.5663265 | 0.2609507 | -2.18E+00 | 1.00E+00 |
| **H/ti/-L/tu/** | **-2.10E+00** | **0.1221489** | **0.278097** | **-7.56E+00** | **5.47E-12***** |
| H/ti/-R/tu/ | -3.79E-01 | 0.6843112 | 0.2637397 | -1.44E+00 | 1.00E+00 |
| **H/ti/-F/ni/** | **-1.80E+00** | **0.1646765** | **0.2688114** | **-6.71E+00** | **2.64E-09***** |
| H/ti/-H/ni/ | -6.30E-01 | 0.5326166 | 0.2602814 | -2.42E+00 | 1.00E+00 |
| **H/ti/-L/ni/** | **-1.60E+00** | **0.2016466** | **0.2643318** | **-6.06E+00** | **1.88E-07***** |
| H/ti/-R/ni/ | -3.69E-02 | 0.9637487 | 0.2717647 | -1.36E-01 | 1.00E+00 |
| **H/ti/-F/nu/** | **-2.18E+00** | **0.1125392** | **0.2812551** | **-7.77E+00** | **1.09E-12***** |
| H/ti/-H/nu/ | -6.91E-01 | 0.5012315 | 0.2597296 | -2.66E+00 | 1.00E+00 |
| **H/ti/-L/nu/** | **-1.77E+00** | **0.1705069** | **0.2679426** | **-6.60E+00** | **5.51E-09***** |
| H/ti/-R/nu/ | -9.00E-01 | 0.4065934 | 0.258653 | -3.48E+00 | 6.84E-02^.^ |
| **L/ti/-R/ti/** | **1.33E+00** | **3.7761836** | **0.2579556** | **5.15E+00** | **3.52E-05***** |
| L/ti/-F/tu/ | -1.07E-01 | 0.8981644 | 0.2676818 | -4.01E-01 | 1.00E+00 |
| **L/ti/-H/tu/** | **1.24E+00** | **3.4390244** | **0.2566957** | **4.81E+00** | **2.03E-04***** |
| L/ti/-L/tu/ | -2.99E-01 | 0.7417504 | 0.2741084 | -1.09E+00 | 1.00E+00 |
| **L/ti/-R/tu/** | **1.42E+00** | **4.1554878** | **0.2595305** | **5.49E+00** | **5.51E-06***** |
| L/ti/-F/ni/ | 4.16E-15 | 1 | 0.2646829 | 1.57E-14 | 1.00E+00 |
| **L/ti/-H/ni/** | **1.17E+00** | **3.2343206** | **0.2560154** | **4.58E+00** | **6.18E-04***** |
| L/ti/-L/ni/ | 2.03E-01 | 1.2245011 | 0.2601322 | 7.79E-01 | 1.00E+00 |
| **L/ti/-R/ni/** | **1.77E+00** | **5.8523748** | **0.2676818** | **6.60E+00** | **5.57E-09***** |
| L/ti/-F/nu/ | -3.81E-01 | 0.6833959 | 0.2773119 | -1.37E+00 | 1.00E+00 |
| **L/ti/-H/nu/** | **1.11E+00** | **3.0437342** | **0.2554543** | **4.36E+00** | **1.79E-03**** |
| L/ti/-L/nu/ | 3.48E-02 | 1.0354052 | 0.2638005 | 1.32E-01 | 1.00E+00 |
| L/ti/-R/nu/ | 9.04E-01 | 2.4690432 | 0.2543597 | 3.55E+00 | 5.17E-02^.^ |
| **R/ti/-F/tu/** | **-1.44E+00** | **0.2378498** | **0.2610318** | **-5.50E+00** | **5.12E-06***** |
| R/ti/-H/tu/ | -9.35E-02 | 0.9107143 | 0.2497535 | -3.74E-01 | 1.00E+00 |
| **R/ti/-L/tu/** | **-1.63E+00** | **0.1964286** | **0.2676182** | **-6.08E+00** | **1.62E-07***** |
| R/ti/-R/tu/ | 9.57E-02 | 1.1004464 | 0.2526662 | 3.79E-01 | 1.00E+00 |
| **R/ti/-F/ni/** | **-1.33E+00** | **0.2648176** | **0.2579556** | **-5.15E+00** | **3.52E-05***** |
| R/ti/-H/ni/ | -1.55E-01 | 0.8565051 | 0.2490542 | -6.22E-01 | 1.00E+00 |
| **R/ti/-L/ni/** | **-1.13E+00** | **0.3242695** | **0.2532841** | **-4.45E+00** | **1.19E-03**** |
| R/ti/-R/ni/ | 4.38E-01 | 1.549812 | 0.2610318 | 1.68E+00 | 1.00E+00 |
| **R/ti/-F/nu/** | **-1.71E+00** | **0.1809753** | **0.2708985** | **-6.31E+00** | **3.79E-08***** |
| R/ti/-H/nu/ | -2.16E-01 | 0.8060345 | 0.2484774 | -8.68E-01 | 1.00E+00 |
| **R/ti/-L/nu/** | **-1.29E+00** | **0.2741935** | **0.2570502** | **-5.03E+00** | **6.54E-05***** |
| R/ti/-R/nu/ | -4.25E-01 | 0.6538462 | 0.2473518 | -1.72E+00 | 1.00E+00 |
| **F/tu/-H/tu/** | **1.34E+00** | **3.8289474** | **0.2597869** | **5.17E+00** | **3.22E-05***** |
| F/tu/-L/tu/ | -1.91E-01 | 0.8258514 | 0.2770052 | -6.91E-01 | 1.00E+00 |
| **F/tu/-R/tu/** | **1.53E+00** | **4.6266447** | **0.2625883** | **5.83E+00** | **7.38E-07***** |
| F/tu/-F/ni/ | 1.07E-01 | 1.1133819 | 0.2676818 | 4.01E-01 | 1.00E+00 |
| **F/tu/-H/ni/** | **1.28E+00** | **3.6010338** | **0.2591146** | **4.94E+00** | **1.04E-04***** |
| F/tu/-L/ni/ | 3.10E-01 | 1.3633373 | 0.2631829 | 1.18E+00 | 1.00E+00 |
| **F/tu/-R/ni/** | **1.87E+00** | **6.515928** | **0.2706474** | **6.93E+00** | **5.93E-10***** |
| F/tu/-F/nu/ | -2.73E-01 | 0.7608806 | 0.2801757 | -9.75E-01 | 1.00E+00 |
| **F/tu/-H/nu/** | **1.22E+00** | **3.3888385** | **0.2585603** | **4.72E+00** | **3.20E-04***** |
| F/tu/-L/nu/ | 1.42E-01 | 1.1528014 | 0.2668093 | 5.33E-01 | 1.00E+00 |
| **F/tu/-R/nu/** | **1.01E+00** | **2.7489879** | **0.2574788** | **3.93E+00** | **1.17E-02*** |
| **H/tu/-L/tu/** | **-1.53E+00** | **0.2156863** | **0.266404** | **-5.76E+00** | **1.16E-06***** |
| H/tu/-R/tu/ | 1.89E-01 | 1.2083333 | 0.2513797 | 7.53E-01 | 1.00E+00 |
| **H/tu/-F/ni/** | **-1.24E+00** | **0.2907801** | **0.2566957** | **-4.81E+00** | **2.03E-04***** |
| H/tu/-H/ni/ | -6.14E-02 | 0.9404762 | 0.247749 | -2.48E-01 | 1.00E+00 |
| **H/tu/-L/ni/** | **-1.03E+00** | **0.3560606** | **0.2520008** | **-4.10E+00** | **5.67E-03**** |
| H/tu/-R/ni/ | 5.32E-01 | 1.7017544 | 0.2597869 | 2.05E+00 | 1.00E+00 |
| **H/tu/-F/nu/** | **-1.62E+00** | **0.1987179** | **0.2696991** | **-5.99E+00** | **2.83E-07***** |
| H/tu/-H/nu/ | -1.22E-01 | 0.8850575 | 0.2471692 | -4.94E-01 | 1.00E+00 |
| **H/tu/-L/nu/** | **-1.20E+00** | **0.3010753** | **0.2557858** | **-4.69E+00** | **3.66E-04***** |
| H/tu/-R/nu/ | -3.31E-01 | 0.7179487 | 0.2460377 | -1.35E+00 | 1.00E+00 |
| **L/tu/-R/tu/** | **1.72E+00** | **5.6022727** | **0.2691365** | **6.40E+00** | **2.08E-08***** |
| L/tu/-F/ni/ | 2.99E-01 | 1.3481625 | 0.2741084 | 1.09E+00 | 1.00E+00 |
| **L/tu/-H/ni/** | **1.47E+00** | **4.3603896** | **0.2657485** | **5.54E+00** | **4.09E-06***** |
| L/tu/-L/ni/ | 5.01E-01 | 1.6508264 | 0.2697167 | 1.86E+00 | 1.00E+00 |
| **L/tu/-R/ni/** | **2.07E+00** | **7.8899522** | **0.2770052** | **7.46E+00** | **1.21E-11***** |
| L/tu/-F/nu/ | -8.19E-02 | 0.9213287 | 0.286322 | -2.86E-01 | 1.00E+00 |
| **L/tu/-H/nu/** | **1.41E+00** | **4.1034483** | **0.265208** | **5.32E+00** | **1.38E-05***** |
| L/tu/-L/nu/ | 3.34E-01 | 1.3958944 | 0.2732564 | 1.22E+00 | 1.00E+00 |
| **L/tu/-R/nu/** | **1.20E+00** | **3.3286713** | **0.2641538** | **4.55E+00** | **7.21E-04***** |
| **R/tu/-F/ni/** | **-1.42E+00** | **0.2406456** | **0.2595305** | **-5.49E+00** | **5.51E-06***** |
| R/tu/-H/ni/ | -2.51E-01 | 0.7783251 | 0.250685 | -1.00E+00 | 1.00E+00 |
| **R/tu/-L/ni/** | **-1.22E+00** | **0.2946708** | **0.2548878** | **-4.79E+00** | **2.23E-04***** |
| R/tu/-R/ni/ | 3.42E-01 | 1.4083485 | 0.2625883 | 1.30E+00 | 1.00E+00 |
| **R/tu/-F/nu/** | **-1.81E+00** | **0.1644562** | **0.2723985** | **-6.63E+00** | **4.67E-09***** |
| R/tu/-H/nu/ | -3.11E-01 | 0.7324614 | 0.2501119 | -1.24E+00 | 1.00E+00 |
| **R/tu/-L/nu/** | **-1.39E+00** | **0.2491657** | **0.2586306** | **-5.37E+00** | **1.05E-05***** |
| R/tu/-R/nu/ | -5.21E-01 | 0.5941645 | 0.2489938 | -2.09E+00 | 1.00E+00 |
| **F/ni/-H/ni/** | **1.17E+00** | **3.2343206** | **0.2560154** | **4.58E+00** | **6.18E-04***** |
| F/ni/-L/ni/ | 2.03E-01 | 1.2245011 | 0.2601322 | 7.79E-01 | 1.00E+00 |
| **F/ni/-R/ni/** | **1.77E+00** | **5.8523748** | **0.2676818** | **6.60E+00** | **5.57E-09***** |
| F/ni/-F/nu/ | -3.81E-01 | 0.6833959 | 0.2773119 | -1.37E+00 | 1.00E+00 |
| **F/ni/-H/nu/** | **1.11E+00** | **3.0437342** | **0.2554543** | **4.36E+00** | **1.79E-03**** |
| F/ni/-L/nu/ | 3.48E-02 | 1.0354052 | 0.2638005 | 1.32E-01 | 1.00E+00 |
| F/ni/-R/nu/ | 9.04E-01 | 2.4690432 | 0.2543597 | 3.55E+00 | 5.17E-02^.^ |
| **H/ni/-L/ni/** | **-9.71E-01** | **0.3785961** | **0.2513078** | **-3.86E+00** | **1.51E-02*** |
| H/ni/-R/ni/ | 5.93E-01 | 1.8094604 | 0.2591146 | 2.29E+00 | 1.00E+00 |
| **H/ni/-F/nu/** | **-1.55E+00** | **0.211295** | **0.2690516** | **-5.78E+00** | **1.03E-06***** |
| H/ni/-H/nu/ | -6.07E-02 | 0.9410738 | 0.2464625 | -2.46E-01 | 1.00E+00 |
| **H/ni/-L/nu/** | **-1.14E+00** | **0.3201307** | **0.255103** | **-4.46E+00** | **1.09E-03**** |
| H/ni/-R/nu/ | -2.70E-01 | 0.7633885 | 0.2453277 | -1.10E+00 | 1.00E+00 |
| **L/ni/-R/ni/** | **1.56E+00** | **4.7793953** | **0.2631829** | **5.94E+00** | **3.79E-07***** |
| L/ni/-F/nu/ | -5.83E-01 | 0.5581015 | 0.2729718 | -2.14E+00 | 1.00E+00 |
| **L/ni/-H/nu/** | **9.11E-01** | **2.4856933** | **0.2507362** | **3.63E+00** | **3.83E-02*** |
| L/ni/-L/nu/ | -1.68E-01 | 0.8455731 | 0.2592343 | -6.47E-01 | 1.00E+00 |
| L/ni/-R/nu/ | 7.01E-01 | 2.0163666 | 0.2496208 | 2.81E+00 | 6.75E-01 |
| **R/ni/-F/nu/** | **-2.15E+00** | **0.1167724** | **0.2801757** | **-7.66E+00** | **2.43E-12***** |
| R/ni/-H/nu/ | -6.54E-01 | 0.5200853 | 0.2585603 | -2.53E+00 | 1.00E+00 |
| **R/ni/-L/nu/** | **-1.73E+00** | **0.1769205** | **0.2668093** | **-6.49E+00** | **1.15E-08***** |
| R/ni/-R/nu/ | -8.63E-01 | 0.4218874 | 0.2574788 | -3.35E+00 | 1.09E-01 |
| **F/nu/-H/nu/** | **1.49E+00** | **4.4538376** | **0.2685177** | **5.56E+00** | **3.61E-06***** |
| F/nu/-L/nu/ | 4.15E-01 | 1.5150884 | 0.2764698 | 1.50E+00 | 1.00E+00 |
| **F/nu/-R/nu/** | **1.28E+00** | **3.6129032** | **0.2674766** | **4.80E+00** | **2.13E-04***** |
| **H/nu/-L/nu/** | **-1.08E+00** | **0.340176** | **0.2545399** | **-4.24E+00** | **3.09E-03**** |
| H/nu/-R/nu/ | -2.09E-01 | 0.8111888 | 0.2447422 | -8.55E-01 | 1.00E+00 |
| L/nu/-R/nu/ | 8.69E-01 | 2.3846154 | 0.2534413 | 3.43E+00 | 8.24E-02^.^ |

**Table S4.15**

*Post-hoc Power of the Valence models in Nonce word*

| Model | Predictor | Effect size/Odds | *Power* |
| --- | --- | --- | --- |
| Valence ~ Tone | Intercept (Tone F) | 2.3913175 | 1 |
|  | H vs. F | 0.2509094 | 1 |
|  | L vs. F | 1.0000036 | 1 |
|  | R vs. F | 0.2448151 | 1 |
| Valence ~ Vowel | Intercept (Vowel /i/) | 1.000165 | 1 |
|  | /u/ vs. /i/ | 1.363605 | 1 |
| Valence ~ Tone * Vowel | Intercept (ToneF:Vowel/i/) | 1.7416325 | 1 |
|  | H vs. F | 0.6155647 | 1 |
|  | L vs. F | 0.3612886 | 1 |
|  | R vs. F | 0.8323272 | 1 |
|  | /u/ vs. /i/ | 1.6180304 | 1 |
|  | H:/u/ vs. F:/i/ | 0.4665391 | 1 |
|  | L:/u/ vs. F:/i/ | 0.7809175 | 1 |
|  | R:/u/ vs. F:/i/ | 0.8993972 | 1 |
| Valence ~ Consonant | Intercept (Consonant /t/) | 1.4257893 | 1 |
|  | /n/ vs. /t/ | 0.7647636 | 1 |
| Valence ~ Tone * Consonant | Intercept (ToneF:Consonant/t/) | 1.8723404 | 1 |
|  | H vs. F | 0.280624 | 1 |
|  | L vs. F | 1.1045971 | 1 |
|  | R vs. F | 0.2626193 | 1 |
|  | /n/ vs. /t/ | 1.55583 | 1 |
|  | H:/n/ vs. F:/t/ | 0.8671398 | 1 |
|  | L:/n/ vs. F:/t/ | 0.8080207 | 1 |
|  | R:/n/ vs. F:/t/ | 0.9408171 | 1 |
| Valence ~ Vowel * Consonant | Intercept (Vowel/i/:Consonant/t/) | 0.9555645 | 1 |
|  | /u/ vs. /i/ | 1.3261824 | 1 |
|  | /n/ vs. /t/ | 1.0955365 | 1 |
|  | /nu/ vs. /ti/ | 1.0581173 | 1 |
| Valence ~ Tone * Vowel * Consonant | Intercept (ToneF:Vowel/i/:Consonant/t/) | 1.5471698 | 1 |
|  | H vs. F | 0.2440269 | 1 |
|  | L vs. F | 1.481856 | 1 |
|  | R vs. F | 0.3924216 | 1 |
|  | /u/ vs. /i/ | 1.6498716 | 1 |
|  | /n/ vs. /t/ | 1.481856 | 1 |
|  | H:/u/ vs. F:/i/ | 1.0702443 | 1 |
|  | L:/u/ vs. F:/i/ | 0.8171317 | 1 |
|  | R:/u/ vs. F:/i/ | 0.5507835 | 1 |
|  | H:/n/ vs. F:/t/ | 1.2670078 | 1 |
|  | L:/n/ vs. F:/t/ | 0.5511056 | 1 |
|  | R:/n/ vs. F:/t/ | 0.4354266 | 1 |
|  | /nu/ vs. /ti/ | 0.8869058 | 1 |
|  | H:/nu/ vs. F:/ti/ | 0.6785248 | 1 |
|  | L:/nu/ vs. F:/ti/ | 0.9890748 | 1 |
|  | R:/nu/ vs. F:/ti/ | 2.9409989 | 1 |

# **5 Intraclass Correlation/Reliability Assessment on Participants Ratings**

To assess the degree of agreement among participants in judging the emotional valence of the stimuli (0 = negative, 1 = positive), inter-rater reliability was estimated using two complementary statistics: the Intraclass Correlation Coefficient (ICC) and the Fleiss’ Kappa (κ). The ICC was computed using a two-way random-effects model, treating both raters and stimuli as random samples from larger populations. Two forms of ICC were calculated: (1) ICC (c, 1; consistency, single measures), which reflects the consistency of ratings across raters, even if some raters tend to use the “positive” label more often than others; (2) ICC (A, k; agreement, average measures), which reflects the absolute agreement among raters’ average ratings for each stimulus. Although ICC assumes continuous data, it was used here as an approximate index of rating consistency, following previous research applying ICC to binary data when the focus is on relative agreement patterns (e.g., Koo & Li, 2016).

To obtain a categorical measure that directly accounts for chance agreement, Fleiss’ Kappa was also computed. Fleiss’ κ is specifically designed for nominal or binary responses and quantifies the extent to which raters agree beyond what would be expected by chance (e.g., Sim & Wright, 2005).

Across all datasets, ICC (A,k) values were consistently high (.878 - .965), indicating excellent reliability of aggregated ratings. Although individual consistency was low (ICC(c,1) = .059 - .183), this is expected for binary judgments and reflects individual variability rather than measurement instability. Fleiss’ κ values were slightly negative and nonsignificant, a typical outcome for dichotomous data with skewed distributions. Together, these results confirm that, despite discrepancies between indices, the aggregated participant ratings are highly reliable and robust.

**Table S5.1**

*Reliability of Participant Ratings for Arousal and Valence in V and CVL Datasets*

| Dataset | Measure | Items | Raters | Index | Estimate | 95% CI | F(df1, df2) | p |
| --- | --- | --- | --- | --- | --- | --- | --- | --- |
| V | Arousal | 8 | 121 | ICC(c,1) | 0.183 | [.085, .489] | 28.2 (7, 840) | <.001 |
|  |  |  |  | ICC(A,121) | 0.965 | [.918, .991] | 28.2 (7, 960) | <.001 |
|  |  |  |  | Fleiss’ κ | -0.0847 | — | z = -0.832 | 0.405 |
|  | Valence | 8 | 135 | ICC(c,1) | 0.101 | [.043, .329] | 16.2 (7, 938) | <.001 |
|  |  |  |  | ICC(A,135) | 0.937 | [.855, .985] | 16.2 (7, 1070) | <.001 |
|  |  |  |  | Fleiss’ κ | -0.0667 | — | z = -0.730 | 0.465 |
| CVL | Arousal | 16 | 121 | ICC(c,1) | 0.0592 | [.030, .140] | 8.61 (15, 1800) | <.001 |
|  |  |  |  | ICC(A,121) | 0.878 | [.777, .949] | 8.61 (15, 1837) | <.001 |
|  |  |  |  | Fleiss’ κ | -0.0535 | — | z = -0.937 | 0.349 |
|  | Valence | 16 | 135 | ICC(c,1) | 0.122 | [.067, .256] | 19.8 (15, 2010) | <.001 |
|  |  |  |  | ICC(A,135) | 0.948 | [.904, .978] | 19.8 (15, 2100) | <.001 |
|  |  |  |  | Fleiss’ κ | -0.0407 | — | z = -0.810 | 0.418 |
